# Engineering Yeast Hexokinase 2 for Improved Tolerance Toward Xylose-Induced Inactivation

**DOI:** 10.1371/journal.pone.0075055

**Published:** 2013-09-06

**Authors:** Basti Bergdahl, Anders G. Sandström, Celina Borgström, Tarinee Boonyawan, Ed W. J. van Niel, Marie F. Gorwa-Grauslund

**Affiliations:** Division of Applied Microbiology, Department of Chemistry, Lund University, Lund, Sweden; University of Strathclyde, United States of America

## Abstract

Hexokinase 2 (Hxk2p) from *Saccharomyces cerevisiae* is a bi-functional enzyme being both a catalyst and an important regulator in the glucose repression signal. In the presence of xylose Hxk2p is irreversibly inactivated through an autophosphorylation mechanism, affecting all functions. Consequently, the regulation of genes involved in sugar transport and fermentative metabolism is impaired. The aim of the study was to obtain new Hxk2p-variants, immune to the autophosphorylation, which potentially can restore the repressive capability closer to its nominal level. In this study we constructed the first condensed, rationally designed combinatorial library targeting the active-site in Hxk2p. We combined protein engineering and genetic engineering for efficient screening and identified a variant with Phe159 changed to tyrosine. This variant had 64% higher catalytic activity in the presence of xylose compared to the wild-type and is expected to be a key component for increasing the productivity of recombinant xylose-fermenting strains for bioethanol production from lignocellulosic feedstocks.

## Introduction

The yeast *Saccharomyces cerevisiae* has three structural genes encoding enzymes that catalyse the phosphorylation of glucose to glucose 6-phosphate: *HXK1*, *HXK2* and *GLK1* [1]. *HXK1* and *HXK2* encode hexokinases which, in addition to glucose, also can phosphorylate fructose and mannose. These two proteins share a protein identity of 77%. *GLK1* encodes a glucokinase which has an identity of 38% with the two hexokinases and lacks the ability to phosphorylate fructose [1]. During growth on fermentable carbon sources hexokinase 2 (Hxk2p) provides the main sugar-phosphorylating capability [2]. When the conditions change to non-fermentable carbon sources the *HXK2* gene becomes repressed and the *HXK1* and *GLK1* genes are rapidly de-repressed [3].

Hexokinase 2 has a bi-functional role in yeast metabolism; in addition to its catalytic function it also has an important regulatory function in the glucose catabolite repression mechanism [4], [5], being an essential Snf1p-antagonist and co-repressor with Mig1p. The Snf1p pathway controls the expression of genes required for growth on less preferred fermentable carbon sources (e.g. galactose, sucrose and maltose) and non-fermentable carbon sources like glycerol and ethanol [6]. Snf1p is activated upon glucose depletion through phosphorylation by three kinases: Sak1p, Elm1p and Tos3p [7]. Addition of glucose effectively inactivates Snf1p by stimulating the activity of the Glc7p/Reg1p phosphatase complex, located in the cytosol [8]. Hxk2p is essential in this deactivation mechanism by enabling Glc7p/Reg1p to dephosphorylate Snf1p [8]. Hence, in a *hxk2*Δ mutant, Snf1p never becomes dephosphorylated and target genes are constitutively expressed, even at high concentrations of glucose [9], [10]. In the presence of glucose Hxk2p is transported into the nucleus by the karyopherin α/β carrier complex Kap60p/Kap95p [11], [12]. Inside the nucleus, Hxk2p interacts with Mig1p and forms a DNA-binding repressor complex [13], [14]. Mig1p is the transcription factor responsible for the repression of genes needed for utilization of alternative fermentable carbon sources [6]. The Hxk2p/Mig1p complex binds to the Mig1p-recognition sites upstream of target genes and in the case of *SUC2* repression, Hxk2p also interacts with Med8p which supposedly hinders the activation of RNA polymerase [4], [5], [15]. In de-repressing conditions active Snf1p is transported into the nucleus and phosphorylates Mig1p leading to its transport out from the nucleus [16]. Active Snf1p can also phosphorylate Hxk2p at residue Ser15 (numbered according to the translated mRNA sequence) which prevents Hxk2p from entering the nucleus [11]. In the absence of Hxk2p, Mig1p is constitutively phosphorylated in the nucleus and transported out with loss of repressive capacity as a result [13]. Transcriptional analysis of a *hxk2Δ*-strain showed significant upregulation of genes with binding sites for Mig1p and/or Cat8p (e.g. *FBP1*, *MDH2*, *MDH3, MLS1*, *ICL1*, *IDP2* and *PCK1*) [17], [18]. This confirms the significant role of Hxk2p in the Snf1p circuit of the glucose signalling mechanism. In addition, Hxk2p also participates in the control of genes encoding sugar transporters [17], [18], [19]. The presence of Hxk2p is required to fully induce *HXT1* at high glucose concentrations and *HXT2* and *HXT4* at low concentrations [19]. Hxk2p is also needed to repress the expression of *HXT2* and *HXT4* at high glucose concentration [19]. Finally, a specific role for Hxk2p in modulating mitochondrial cytochrome content and respiratory activity has been suggested [20]. This is supported by a diminished Crabtree effect in *hxk2*Δ mutants, resulting in a nearly complete respiratory metabolism at high glucose concentration [18]. In light of these findings, the Hxk2p protein can be regarded as a global glycolytic regulator that is essential for mediating the complete glucose repression signal.

Early studies on glucose catabolite repression revealed that xylose can induce a decrease in the glucose phosphorylating activity [2]. *In vitro* experiments confirmed that Hxk2p becomes irreversibly inactivated by xylose through an autophosphorylation mechanism in the presence of ATP [21]. The target residue for the autophosphorylation was later identified as Ser158 situated in the active site [22]. The exchange of this amino acid indeed abolished the autophosphorylation but also severely reduced catalytic activity [22]. The induced inactivation by xylose also caused an increase in invertase activity (encoded by *SUC2*), demonstrating that both the catalytic and regulatory functions of Hxk2p are affected by the modification [2]. Normally the catalytic and regulatory functions operate independently through different parts of the protein [14], indicating that the phosphorylation of Ser158 has a profound influence on Hxk2p functionality. Alteration of Ser158 significantly reduces the ability of Hxk2p to stimulate the Ras/cAMP signal [23], [24], a signal that induces a fast and comprehensive modulation of the transcriptional organization with the aim to enhance cellular metabolism and growth [25].

In the production of bioethanol from lignocellulosic biomass, an efficient conversion of xylose is of great importance to increase the economic feasibility of the process [26]. *S. cerevisiae* lacks the ability to ferment xylose and requires extensive genetic engineering to obtain such a trait [27]. However, xylose is not recognised as a fermentable carbon source by this yeast, as shown in several transcription analyses [28], [29], [30] and profiling of intracellular metabolite concentrations [31], [32]. Due to the distinguished role of Hxk2p in glucose repression signalling we hypothesised that the inactivation by xylose leads to a reduced capability to regulate expression of genes involved in sugar transport and fermentative metabolism. The aim of the current study was to identify new variants of Hxk2p, immune to autophosphorylation by xylose, which were expected to increase the sugar consumption rate during fermentation of a glucose/xylose mixture by restoring the repressive capability closer to its nominal level, thus increasing ethanol productivity. The over-expression of one such variant did indeed result in a faster xylose uptake rate and a faster initial growth rate on xylose but other regulatory factors, normally induced by glucose, are still needed to significantly increase the ethanol productivity from xylose.

## Materials and Methods

### Strains and culture conditions

Plasmids and yeast strains used are listed in Table 1. Frozen yeast strains were recovered on solid YPD or YPGal plates (10 g L^−1^ yeast extract, 20 g L^−1^ peptone, 20 g L^−1^ glucose or galactose, 15 g L^−1^ agar) for two days at 30°C. Yeast cultures were grown in liquid YPD or YPGal medium for 12–16 h at 30°C and 180 rpm in an orbital shaker. Competent yeast cells were prepared and transformed according to the high efficiency protocol [33]. Transformants were selected on solid Yeast Nitrogen Base (YNB) medium (6.7 g L^−1^ YNB without amino acids, 15 g L^−1^ agar) with 20 g L^−1^ glucose or galactose as carbon source. When required, uracil and leucine were supplemented at 50 mg L^−1^ and 220 mg L^−1^, respectively, and the antibiotics geniticin and aureobasidin were supplemented at 200 mg L^−1^ and 0.15 mg L^−1^, respectively. In shake flask cultivations, YNB medium contained 50 mM potassium hydrogen phthalate buffer at pH 5.5. Anaerobic continuous cultivations were made in a defined mineral medium [34] using 5 g L^−1^ glucose and 50 g L^−1^ xylose or 15 g L^−1^ glucose with or without 30 g L^−1^ xylose in the feed reservoir. All media used for anaerobic cultivations were supplemented with Tween 80 (400 mg L^−1^) and ergosterol (10 mg L^−1^).

**Table 1:** Strains and plasmids used in this study.

Name	Relevant genotype^a^	Reference
**Plasmids**		
p424	*TRP1*	[54]
p426	*URA3*	[54]
pUG6	LoxP-*KanMX*-LoxP	[55]
pUG6AUR	LoxP-*AUR1*-LoxP	[56]
pUG62AUR-HXK1USDS	*HXK1*(−294–−21 bp), *HXK1*(+1780–+1999 bp), LoxP-*AUR1*-LoxP	This study
YIplac128	*LEU2*	[57]
YIplac211	*URA3*	[57]
YIpDR1	YIplac128:*TDH3*p-*GXF1*-*CYC1*t	[58]
YIpDR7	YIplac211:*TDH3*p-*XYL1*(N272D)-*ADH1*t; *PGK1*p-*XYL2*-*PGK1*t	[46]
YIpBB5	YIplac128:*HXK2*p-*HXK2*-*HXK2*t	This study
YIpBB8	YIplac128:*TDH3*p-*HXK2*-*CYC1*t	This study
YIpBB8Y	YIplac128:*TDH3*p-*HXK2*(F159Y)-*CYC1*t	This study
**Deletion cassettes**		
*HXK2*	*HXK2*(−500–−1 bp)-*TRP1*-*HXK2*(+1461–+1960 bp)	This study
*HXK1*	*HXK1*(−1000–−451 bp)-*URA3*-*HXK1*(+2040–+2353 bp)	This study
*GLK1*	*GLK1*(−673–−121 bp)-LoxP-*KanMX*-LoxP-*GLK1*(+1858–+2359 bp)	This study
**Yeast strains**		
TMB3042	CEN.PK2-1C; *gre3*-Δ; *his3*::*PGK1*p-*XKS1*-*PGK1*t, *HIS3*; *tal1*::*PGK1*p-*TAL1*-*PGK1*t; *tkl1*::*PGK1*p-*TKL1*-*PGK1*t; *rki1*::*PGK1*p-*RKI1*-*PGK1*t; *rpe1*::*PGK1*p-*RPE1*-*PGK1*t; *trp1*, *ura3*, *leu2*	[45]
TMB3460	TMB3042; *hxk2*-Δ::*TRP1*; *ura3*, *leu2*	This study
TMB3461	TMB3460; *hxk1*-Δ*1*::*URA3*; *leu2*	This study
TMB3462	TMB3461; *glk1*-Δ::LoxP-*KanMX*-LoxP; *leu2*	This study
TMB3463	TMB3462; *hxk1*-Δ*2*::pUG62AUR-HXK1USDS; *leu2*	This study
TMB3466	TMB3463; *leu2*::YIpBB8	This study
TMB3467	TMB3463; *leu2*::YIpBB8Y	This study
TMB3490	TMB3460; *ura3*::YIpDR7; *leu2*	This study
TMB3492	TMB3490; *leu2*::YIpBB8	This study
TMB3493	TMB3490; *leu2*::YIpBB8Y	This study

a Sequence numbering for some constructs are numbered in relation to the first base in the ATG starting codon of the ORF in question.

*E. coli* strain NEB5α (New England Biolabs, USA) was used for sub-cloning of plasmid DNA. Heat shock competent cells were prepared according to the Inoue method [35] and transformed according to the supplier's instructions. Transformants were selected from solid LB plates (5 g L^−1^ yeast extract, 10 g L^−1^ tryptone, 10 g L^−1^ NaCl, 15 g L^−1^ agar, pH 7.0), supplemented with 100 mg L^−1^ of ampicillin, after incubation for 16 h at 37°C. Cultures of transformed cells were grown in liquid LB medium with ampicillin, for 14–16 h at 37°C and 180 rpm.

### Structural analysis of Hxk2p

A homology model of *S. cerevisiae* Hxk2p was generated based on the closed conformation of the *S. cerevisiae* Hxk1p scaffold with a glucose moiety in the active site (PDB: 3B8A_X). The models were generated using SWISS-MODEL [36] by submitting the Hxk2p (GenBank: EIW10681.1) and the later obtained Hxk2p-Y amino acid sequences for automatic homology modelling. Structural analysis of the model was done manually using PyMOL (http://www.pymol.org). Further information regarding the structural analysis is described in the Results section.

### Construction of an *E. coli* library of Hxk2p-variants

The native *HXK2* gene was amplified from genomic DNA of *S. cerevisiae* CEN.PK2-1C. This fragment also contained a 700 bp upstream promoter-sequence and a 300 bp downstream terminator-sequence. The cloned *HXK2* locus was verified by sequencing and the resulting plasmid was named YIpBB5 (Table 1). The cloned *HXK2* locus was used as template for introducing specific mutations using six degenerate primer pairs (Table S3). The primers were designed such that the 3′-end of each region overlapped with the 5′-end of the following region, enabling the use of overlap-extension PCR (OE-PCR) [37] to link all regions together in a combinatorial manner, generating a heterogeneous mix of the complete locus (see Fig. S1). The plasmid library was generated according to the MEGAWHOP protocol [38] in which the heterogeneous DNA mix were used as megaprimers and the YIpBB5 plasmid as template. The newly synthesised plasmids were subsequently cloned in commercial heat shock competent *E. coli* NEB5α and the presence of all mutations introduced were confirmed by sequencing (Fig. S2). Detailed information on the procedures can be found in the Supporting information.

### Construction of the yeast screening strain TMB3463

The genes *HXK2*, *HXK1* and *GLK1* were deleted sequentially from strain TMB3042 (Table 1). The regions used for homologous recombination were amplified from genomic DNA and flanked the intended ORF to ensure complete gene replacement. Three deletion cassettes with different selection markers were created by OE-PCR (Table 1, Table S4). Correct integration of the cassettes was verified by PCR (Fig. S3B) and the new strains were named TMB3460 (*hxk2-Δ*), TMB3461 (*hxk2-Δ hxk1-Δ1*) and TMB3462 (*hxk2-Δ hxk1-Δ1 glk1-Δ*) (Table 1). The deletions were also verified using primers specific for each gene (Table S5). However, this analysis revealed that the *HXK1* gene was still present in the genome even though the deletion cassette was in the correct locus and the strain was expected to be haploid (Fig. S3C). To delete the second copy of *HXK1* a new deletion vector was constructed based on the pUG6AUR plasmid (Table 1). Additional restriction sites were introduced in the pUG6AUR plasmid by ligating a linker (Fig. S4) into the *Sal*I/*Nde*I-digested plasmid, generating plasmid pUG62AUR (Fig. S5). The linker contained recognition sequences for *Kpn*I, *Sma*I, *Sph*I and *Avr*II. Homologous regions flanking the *HXK1* gene were amplified by PCR from genomic DNA (Table S6), generating an upstream sequence of 273 bp (−294–−21 bp) and a downstream sequence of 219 bp (+1780–+1999 bp). The upstream and downstream fragments were digested with *Sph*I/*Avr*II and *Sph*I/*Kpn*I, respectively, and ligated into pUG62AUR generating the deletion vector pUG62AUR-HXK1USDS (Fig. S6). Correct integration of the vector was verified by PCR (Table S7) and enzyme activity measurements confirmed the absence of any glucose phosphorylating activity (Fig. S7). The final screening strain was named TMB3463 (Table 1). Detailed information on these procedures can be found in the Supporting information.

### Large-scale transformation of TMB3463

A single colony of TMB3463 was inoculated in 5 mL YPGal in a 50 mL conical tube and cultivated for 20 h. This pre-culture was used to inoculate 50 mL YPGal in a 500 mL baffled shake flask at OD = 0.1. After 10.5 h of cultivation the cells were in mid-exponential phase and used to inoculate four shake flasks of 1 L containing 100 mL YPGal each at OD = 0.02. After 14.5 h of growth the cultures reached an OD = 1 and were harvested, washed and transformed according the Large-scale PEG/LiAc protocol [39]. The transformation was performed for 90 min at 42°C in an orbital shaker at 65 rpm, using 300 µg of the plasmid library linearized with *Afl*II. After washing the cells, different volumes were spread on solid YNB medium with galactose. The remaining transformants were inoculated in 1.5 L of defined mineral medium [34] with 20 g L^−1^ galactose as carbon source. The propagation was performed in a 2.5 L Braun bioreactor with a stirring rate of 800 rpm and a sparging rate of 1 L air min^−1^. The pH was automatically maintained at 5.5 by addition of 3 M KOH. Silicon antifoam RD emulsion (Dow Corning, USA) was added at a concentration of 0.5 mL L^−1^. After four days colonies were counted on the plates and the total size of the yeast library was estimated to 1.4×10^6^±3% cfu:s, which is 6 times larger than the size required for 100% coverage [40].

### Selection of a new Hxk2p-variant

Screening of the yeast Hxk2p-library for variants with increased resistance to autophosphorylation by xylose was performed in anaerobic glucose-limited continuous cultivation. After propagating the yeast library aerobically on galactose the culture volume was reduced to 1 L, sparging was changed to N_2_-gas at a flow rate of 0.2 L min^−1^ and the stirring rate was set to 200 rpm. The feed reservoir, containing 5 g L^−1^ glucose and 50 g L^−1^ xylose, was supplied at a rate giving a dilution rate of 0.072 h^−1^. After 96 h the dilution rate was increased to 0.40 h^−1^ initiating a washout of all cells. During the washout, samples for OD and HPLC analysis were collected every hour. After 12 h a final sample was collected and diluted to yield single colonies on solid YNB medium with glucose. The plates were incubated for two days and ten random colonies were selected for sequencing of the *HXK2* gene. The ten *HXK2* ORFs were amplified from genomic DNA by PCR using primers HXK2_seq_f (5′-GAA AAG ATT GTA GGA ATA TAA TTC TC-3′) and HXK2_seq_r (5′-CAC ATA ATT AAA AAA AGG GCA CCT TC-3′). Each reaction mixture (50 µL) contained 1X Buffer, 1.5 mM MgCl_2_, 0.2 mM of each dNTP, 0.5 mM of forward and reverse primer and 1 U High Fidelity Phusion Hotstart II Polymerase (Thermo Scientific, USA). The amplification program was as follows: 30 s denaturation at 98°C, 30 cycles of 10 s denaturation at 98°C, 15 s annealing at 58.3°C, 45 s extension at 72°C and 1 cycle of 10 min final extension at 72°C. The PCR-products were purified using GeneJet PCR Purification Kit (Thermo Scientific, USA) and sent for sequencing.

### Construction of strains TMB3466, 3467, 3492 and 3493

The native *HXK2* gene was amplified by PCR from plasmid YIpBB5 using primers HXK2-F1-SpeI (5′- CTA GAA CTA GTA AAT GGT TCA TTT AGG TCC AAA AAA ACC ACA AG-3′) and HXK2-R1-SalI (5′- GAG GTC GAC TTA AGC ACC GAT GAT ACC AAC GG-3′). The amplification program was as follows: 30 s denaturation at 98°C, 30 cycles of 10 s denaturation at 98°C, 30 s annealing at 65°C (−0.5°C decrease per cycle), 1 min extension at 72°C and 1 cycle of 10 min final extension at 72°C. The gene encoding the selected variant Hxk2p(F159Y) was amplified from genomic DNA of one of the selected clones using the same program. The two *HXK2* genes were digested with *Spe*I and *Sal*I and inserted between the *TDH3*-promoter and *CYC1*-terminator in the vector YIpDR1, yielding the two plasmids YIpBB8 and YIpBB8Y (Table 1). The cloned genes were verified by DNA-sequencing.

Strains with a single hexokinase gene were constructed by transforming TMB3463 with YIpBB8 or YIpBB8Y, both linearized with *Cla*I. The new strains were named TMB3466 and TMB3467, respectively (Table 1). Xylose-fermenting strains were constructed by transforming TMB3460 with YIpDR7, linearized with *Eco*RV, generating strain TMB3490. This strain was further transformed with YIpBB8 or YIpBB8Y generating the strains TMB3492 and TMB3493, respectively (Table 1).

### Carbon limited continuous cultivations

The sensitivity of the native Hxk2p and Hxk2p-Y toward xylose was evaluated in anaerobic glucose-limited continuous cultivations. Strains TMB3466 and TMB3467 (Table 1) were pre-grown in YNB medium with glucose until late exponential phase and inoculated into 3 L stirred-tank bioreactors (Applikon, Schiedam, The Netherlands) equipped with ADI 1025 Bio-Console and ADI 1010 Bio-Controller (Applikon, Schiedam, The Netherlands). Cultivations were performed with a working volume of 1 L of defined mineral medium [34] at 30°C. The medium was continuously stirred at 200 rpm and sparged with N_2_-gas at a rate of 0.2 L min^−1^. The pH was automatically maintained at 5.5 by addition of 3 M KOH. The condenser was connected to a Heto CBN 8–30 cooling bath (Jouan Nordic A/S, Allerød, Denmark) and cooled to 4°C. The off-gas was analysed on-line for O_2_, CO_2_ and ethanol using an INNOVA 1313 Fermentation Monitor (LumaSense Technologies A/S, Denmark). Silicon antifoam RD emulsion (Dow Corning, USA) was added at a concentration of 0.2 mL L^−1^. An initial steady-state was established at *D* = 0.21 h^−1^ using a feed reservoir with 15 g L^−1^ glucose. The cells were then exposed to a gradual increase in xylose concentration by changing to a feed reservoir with 15 g L^−1^ glucose and 30 g L^−1^ xylose. Samples were collected after 5 and 7.5 volume changes with each feed composition, as well as at different time points during the accumulation of xylose. Cell samples (5 mL) were collected on ice and centrifuged for 5 min at 4000 rpm and 4°C. Cell pellets for enzyme activity measurements were stored at −80°C until needed. Fermentation experiments were performed in biological duplicates.

### Anaerobic batch fermentations

Cells were pre-grown in YNB medium with glucose until late exponential phase and inoculated into the bioreactor at a concentration of 0.03 g CDW L^−1^. Fermentation experiments were performed in 3 L stirred-tank bioreactors (Applikon, Schiedam, The Netherlands) as described above except that 1 L of 2× YNB with 20 g L^−1^ glucose and 50 g L^−1^ xylose was used as cultivation medium. Fermentation experiments were performed in biological duplicates.

### Enzyme activity assays

Cell-free extracts were prepared by thawing frozen cell pellets on ice and wash once with water. After a centrifugation step of 5 min at 4000 rpm and 4°C, the cells were treated with 250 µL Y-PER (Thermo Scientific, USA) per 100 mg of wet cell pellet according to the suppliers instructions. Protein concentrations were determined with the Coomassie Protein Assay Reagent (Thermo Scientific, USA) using BSA (Thermo Scientific, USA) as standard. Glucose phosphorylating activity was determined in a 1 mL reaction mixture containing: 20 mM HEPES (pH 7.6), 5 mM glucose, 5 mM MgCl_2_, 1 mM NADP, 1 mM ATP and 1.16 U of glucose 6-phosphate dehydrogenase. The formation of NADPH was measured at 340 nm in a cuvette with light path length of 1 cm using a Ultrospec 2100 Pro spectrophotometer (Amersham Biosciences Corp., USA) controlled by computer software SWIFT Reaction Kinetics v. 2.05 (Biochrom Ltd., Cambridge, UK). The absorbance was converted to concentration units using the molar extinction coefficient of NADPH (ε_340 nm_  = 6220 L mol^−1^ cm^−1^). The slope was determined within an interval of at least 1 min, using at least three different dilutions of the cell extract. Only the slopes within the range 0.05–0.2 A min^−1^ were considered acceptable. One unit is defined as the conversion of 1 µmole of glucose per min.

### Biomass determination and analysis of metabolites

Optical density was measured at 620 nm using an Ultrospec 2100 Pro spectrophotometer (Amersham Biosciences Corp., USA). Cell dry weight was measured in triplicate by filtering a known volume of the culture through a pre-weighed nitrocellulose filter with 0.45 µm pore size. The filters were washed with three volumes of water, dried in a microwave oven for eight minutes at 350 W and weighed after equilibrating to room temperature in a desiccator.

Concentrations of glucose, xylose, xylitol, glycerol, acetate and ethanol were analysed by HPLC (Waters, USA). Metabolites were separated using an Aminex HPX-87H ion exchange column (Bio-Rad, USA) at 45°C with 5 mM H_2_SO_4_ as mobile phase at a flow rate of 0.6 mL min^−1^. All compounds were detected with a RID-10A refractive index detector (Shimadzu, Japan).

### Calculation of metabolic rates

Maximum specific consumption rates of glucose and production rates of glycerol, acetate and ethanol were calculated from Equation 1. 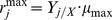
(1)


The biomass yield coefficients (*Y_j_*_/*X*_) were calculated as the slope in a linear regression of cumulative consumption or production of metabolite *j* vs. the biomass concentration. Yield coefficients were calculated from at least eight data points. Maximum specific growth rates on glucose were calculated as the slope in a linear regression of ln(CDW) vs. time from four data points. Due to the non-exponential growth on xylose, Equation 1 cannot be used for calculating specific rates. The maximum specific consumption rates of xylose and production rates of xylitol, glycerol, acetate and ethanol during xylose consumption were instead calculated as the slope in linear regression of metabolite concentration divided by biomass concentration vs. time. These rates were calculated using at least four data points from the phase when xylose was the only carbon source. The significance of a difference between two mean values was calculated using a two-tailed t-test assuming equal variance of the two samples. Comparison of mean values from steady state cultures had a degree of freedom of six (*n_1_* = *n_2_* = 4) whereas all other comparisons had two degrees of freedom (*n_1_* = *n_2_* = 2).

## Results

### Amino acid residues potentially influencing the xylose-induced inactivation

The aim of the study was to identify a new variant of Hxk2p which i) is immune to autophosphorylation by xylose, ii) maintains its regulatory capability, and iii) has high catalytic activity in the presence of xylose. Ser158 in the active site is known to be the target for autophosphorylation [22] and modulation of this residue disrupts the catalytic function as well as the regulatory capability [23]. To fulfil the above mentioned criteria we used a focused protein engineering strategy, targeting the active site of Hxk2p.

A homology model of Hxk2p was generated with a glucose moiety in the active site, based on the closed conformation of the *S. cerevisiae* Hxk1p scaffold. The generated model allowed the study of the amino acid residues directly involved in substrate binding. Several amino acids in different regions surrounding the active site were assessed for their mutability. This assessment was based on both the structural features of the amino acids and on their orientation towards the glucose moiety. The initial set of residues included those within a 6 Å radius volume centred on the C-6 of the β-d-glucopyranose structure. The residues with a side-chain orientation pointing away from the active site were, however, removed from the set. The final set of potentially mutable positions contained 13 amino acid residues (Table 2). The chosen degeneracy introduced mutations in each position creating a codon for either one or three alternative amino acids, or the native amino acid (Table 2). With this approach a condensed library was generated. Although limited in diversity, such a focused library design strategy was previously shown to efficiently select for favourable epistatic effects on another enzyme [41]. Often only discreet changes are needed to induce a required adjustment of substrate preference. Thus, the alternative residue(s) were chosen to have chemical properties similar to the wild-type residue, while chain length and size of the residue were varied. Some substitutions, such as Gly271Cys, were assumed to promote residue-residue interactions and cause slight backbone conformation changes. The isoleucine in position 231 was assumed to be a key residue for distinguishing between the binding of d-glucopyranose and d-xylopyranose [42] as it is pointing directly at the C-6 of glucose. Hence, four different residues (including the wild-type residue) were possible at this position. The theoretical combinatorial protein library was calculated to contain 16,384 variants. In practice, a total size of ca. 57,000 cfu:s was obtained after transforming *E. coli* with the plasmid library, which corresponded to a 97% coverage of the theoretical variants [40].

**Table 2:** Mutations included in the condensed Hxk2p library.

#	Selected residue	Alternative residue(s)	Codon degeneracy^a^
1	Ser158	Thr	ASC
2	Phe159	Tyr	TWC
3	Arg173	Lys	ARA
4	Lys176	Arg	ARA
5	Asp211	Glu	GAK
6	Thr212	Ser	ASC
7	Ile231	Asp/Phe/Tyr	WWC
8	Gly233	Ser	RGC
9	Thr234	Ser	ASC
10	Gly235	Ala	GSA
11	Glu269	Asp	GAK
12	Gly271	Cys	KGC
13	Glu302	Asp	GAK

The table lists the amino acid residues chosen for mutagenesis, the alternative residues in each position and the codon degeneracy used.

a Codon degeneracy: S = C or G; W = A or T; R = A or G; K = G or T.

### Identification of a new Hxk2p variant with a single amino acid substitution

Strain TMB3463, which is unable to grow on glucose due to the absence of hexokinase activity, was constructed to couple the screening to the survival of the host on this carbon source. Hence, when transformed with the library only strains carrying catalytically functional enzyme variants could grow on glucose. To select for a variant immune to inactivation by xylose the population had to be exposed to this sugar and it had to be taken up by the cells. Unfortunately, glucose and xylose are taken up by the same transporters [43] and due to the much higher affinity for glucose the concentration of this sugar has to be as low as possible to maintain a high xylose-mediated selection pressure. For this reason glucose-limited continuous cultivation was chosen for the screening. Due to the role of Hxk2p in fermentative metabolism, anaerobic conditions were chosen for the screening to generate an additional selection pressure for a variant with maintained regulatory function.

After transforming strain TMB3463 with the plasmid library the transformants were propagated in aerobic batch cultivation with galactose as carbon source in order to include variants that might have poor glucose phosphorylating capability. When all galactose was consumed the conditions were changed to anaerobiosis and the chemostat was initiated by supplying the feed, containing 5 g L^−1^ glucose and 50 g L^−1^ xylose, at a rate of 1.16 mL min^−1^ giving a dilution rate of 0.072 h^−1^. At this rate it took ca. 56 h to reach a concentration of 49 g L^−1^ of xylose inside the reactor. During this period all variants, which were either unable to phosphorylate glucose or significantly inactivated by xylose, were expected to wash out from the culture. After 96 h of cultivation the dilution rate was increased to 0.40 h^−1^ and being higher than the maximum growth rate under anaerobic conditions, it initiated a washout of all cells. When the optical density was sufficiently low, the largest fraction of the remaining cells was expected to constitute the variants with the highest growth rate.

**Figure 1:**
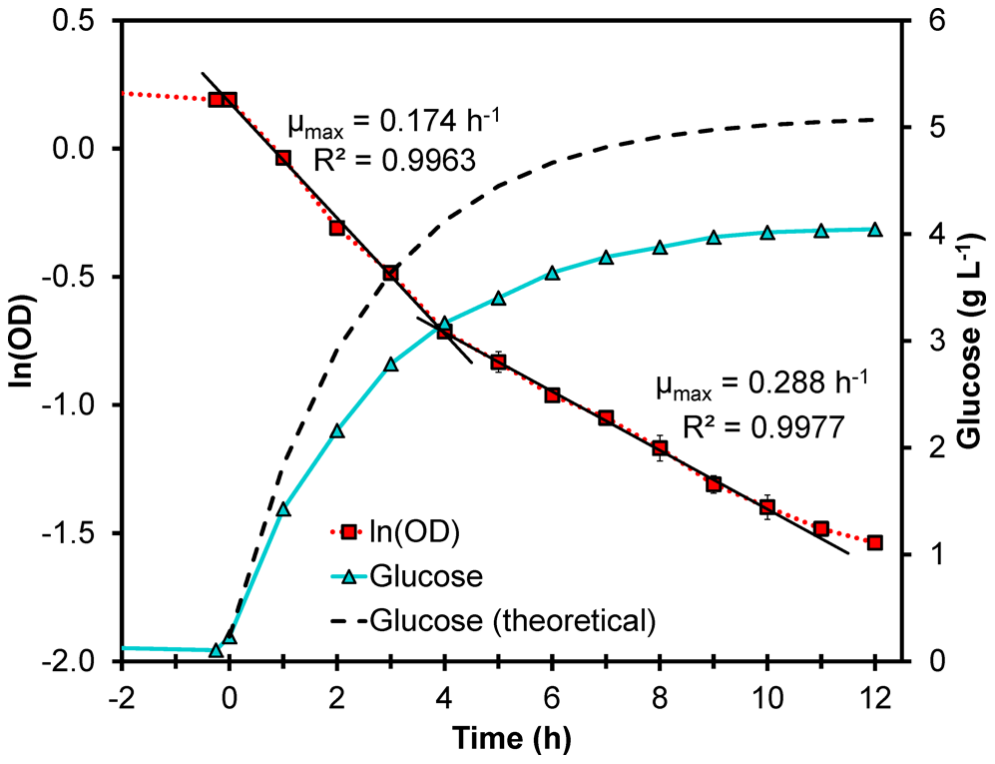
Selection of Hxk2p variants. The selection of Hxk2p variants was performed in anaerobic glucose limited chemostat cultivation with feed containing 5 g L^−1^ glucose and 50 g L^−1^ xylose. After 96 h of continuous cultivation of TMB3463 transformed with the *HXK2*-library at *D* = 0.072 h^−1^, the dilution rate was increased to *D* = 0.40 h^−1^ (indicated as *t* = 0 h). During the washout the specific growth rate was calculated from the equation d ln(OD) d*t* ^−1^ =  *μ*_max_−*D*. The natural logarithm of OD is shown as red squares (▪). The washout profile displays two growth phases and the switching point occurs when the residual glucose concentration (▴) exceeds 3 g L^−1^. After 10 h the glucose concentration stabilized at 4 g L^−1^ which reduced the selection pressure by inhibiting the uptake of xylose and thus slowed down the washout of cells. The measured accumulation of glucose was less than the theoretical (dashed line), calculated according to *S* =  *S*_in_+(*S*_0_−*S*_in_)·e^(−*D*·*t*)^, showing that the consumption was not negligible.

Immediately upon increasing the flow rate the population was able to grow at a rate of 0.17 h^−1^ (Fig. 1). After 4 h the concentration of glucose had increased above 3 g L^−1^ which was enough to reduce the transport of xylose into the cells and as a consequence the growth rate increased to 0.29 h^−1^ (Fig. 1). It took 12 h to reduce the OD from 1.2 to 0.2 at which point the culture was diluted and plated on solid medium to yield single colonies. Ten random colonies were selected for sequencing of the *HXK2* gene. Four of these sequences gave inconclusive results due to the detection of more than one nucleotide in some positions and were thus discarded. The remaining six sequences were of good quality and the majority (4 out of 6) had mutations corresponding to a single amino acid substitution: Phe159Tyr. This variant was designated Hxk2p-Y.

### The Phe159Tyr substitution confers increased resistance to xylose-induced inactivation

**Figure 2:**
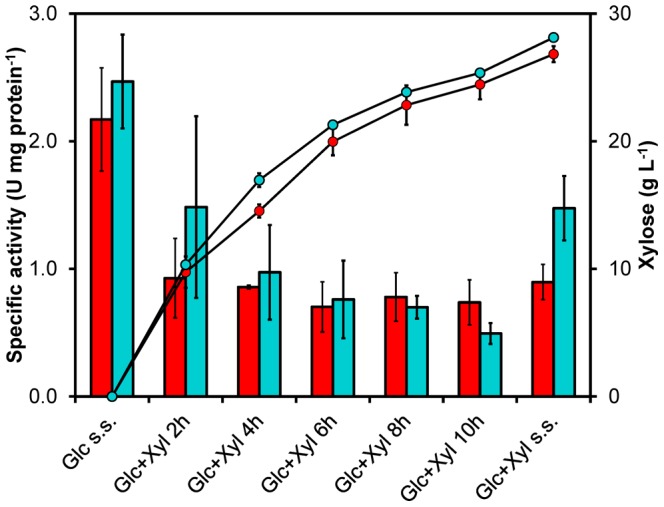
Specific glucose phosphorylating activity during xylose-induced inactivation of Hxk2p-wt and Hxk2p-Y. Specific glucose phosphorylating activity (bars) in strains TMB3466 (Hxk2p-wt) (red colour) and TMB3467 (Hxk2p-Y) (turquoise colour) in anaerobic glucose-limited chemostat cultivations. At steady state (s.s.) on glucose (Glc) the two strains exhibited similar activity but during the accumulation of xylose (Xyl; •) the wild-type enzyme became inhibited faster than the variant. At steady state in the presence of xylose the variant had 64% higher specific activity compared with the wild-type. Specific activities were determined from duplicate biological experiments. At steady state conditions two different samples were collected with at least 2.5 volume changes in between.

To evaluate the effect of the Phe159Tyr substitution on the catalytic activity, two new yeast strains were constructed: TMB3466 (Hxk2p-wt) and TMB3467 (Hxk2p-Y). Both strains lacked all genes encoding hexo- and glucokinases and the expression of the reintroduced gene was controlled by the constitutive *TDH3*-promoter to avoid glucose-controlled gene expression. Both strains were cultivated in anaerobic, glucose-limited chemostat at *D* = 0.22±0.01 h^−1^, first with only glucose in the feed. When steady state had been established the feed was changed to also contain 30 g L^−1^ xylose which led to an accumulation of xylose inside the reactor. At steady state on glucose the two strains exhibited nearly the same specific glucose phosphorylating activity (Fig. 2); hence, the Phe159Tyr substitution had no detrimental effect on the catalytic function. At the second steady state, in the presence of ca. 30 g L^−1^ xylose, the wild-type enzyme lost 59% of the activity whereas the Hxk2p-Y variant only lost 40% (Fig. 2). During the accumulation of xylose there were no statistically significant differences between the two enzymes, although the activity profiles suggested that the wild-type enzyme lost the activity more rapidly than the Hxk2p-Y variant. Still, the 64% higher activity of the variant in the presence of xylose was statistically significant (*P* = 0.007) showing that this single mutation indeed conferred higher resistance to xylose-induced inactivation without decreasing the normal catalytic capability.

### Physiological responses of yeast strains harbouring the Hxk2p-Y variant

In the continuous cultivations of strains TMB3466 (Hxk2p-wt) and TMB3467 (Hxk2p-Y) with only glucose, there were significant differences in the yields of glycerol and biomass (Table 3). Strain TMB3467 had 23% (*P* = 0.04) and 2.6% (*P* = 0.015) higher yields of glycerol and biomass, respectively, compared to TMB3466. Yet, in this condition there was no significant difference in the glucose uptake rate (Table 3). In the presence of xylose, the glucose consumption rate in TMB3466 was reduced by 15% (*P* = 5·10^−5^) while the consumption rate in TMB3467 was only reduced by 5% (*P* = 0.052). Hence, the Hxk2p-Y variant enabled a 15% higher (*P* = 4·10^−4^) specific glucose uptake rate in the presence of xylose compared with Hxk2p-wt. Despite the reduced consumption rate of glucose, TMB3466 did not change its physiology significantly with regard to production rates or product yields when exposed to xylose (Table 3). TMB3467, on the other hand, reduced the yield of biomass by 5.8% (*P* = 6·10^−5^) but increased the ethanol production rate by 6.5% (*P* = 0.048) (Table 3). Hence, TMB3467 had a 8.9% faster (*P* = 0.10) ethanol production rate compared with the control strain in the presence of xylose (Table 3). Furthermore, the specific uptake rate of xylose by TMB3467 was 34% lower (*P* = 0.03) compared to TMB3466 (Table 3). This might be due to improved repression of genes encoding unspecific aldose reductases that can convert xylose to xylitol [44]. In line with this observation, TMB3467 also had a 34% lower (*P* = 0.044) xylitol yield compared to TMB3466 (Table 3), matching the difference in xylose uptake rate very well.

**Table 3:** Specific consumption rates, production rates and yields in anaerobic glucose-limited chemostat cultures of TMB3466 (Hxk2p-wt) and TMB3467 (Hxk2p-Y).

	Glucose steady state		Glucose + xylose steady state	
	TMB3466	TMB3467	TMB3466	TMB3467
**Specific rates (mmol g CDW^−1^ h^−1^)**				
Glucose	−12.9±0.08	−13.2±0.5	−10.9±0.4	−12.5±0.3
Xylose	–	–	−2.75±0.59	−1.82±0.27
Glycerol	2.19±0.03	2.76±0.29	2.34±0.41	2.53±0.04
Acetate	0.15±0.02	0.23±0.09	0.21±0.08	0.20±0.02
Ethanol	20.6±2.43	21.7±1.0	21.3±1.9	23.2±0.6
Xylitol	–	–	0.32±0.09	0.22±0.05
CO_2_ a	21.6±2.44^b^	22.9±0.9	22.4±1.8	24.3±0.6
**Yields (g g sugar^−1^)**				
Glycerol	0.087±0.001	0.107±0.015	0.092±0.022	0.092±0.004
Acetate	0.004±0.001	0.006±0.002	0.006±0.002	0.005±0.000
Ethanol	0.391±0.045	0.402±0.003	0.395±0.010	0.404±0.018
Xylitol	–	–	0.020±0.005	0.013±0.002
Biomass	0.092±0.001	0.094±0.001	0.090±0.007	0.089±0.001
CO_2_	0.410±0.044	0.422±0.002	0.414±0.007	0.423±0.017
Carbon balance (%)	99.1±8.7^b^	103.7±1.5	102.2±1.1	103.1±3.5
Redox balance (%)	98.6±8.6^b^	103.4±1.6	101.9±1.3	102.8±3.5

Values are given as mean ± standard deviation of four measurements from two biological experiments.

a The production rate of CO_2_ was calculated from the production rates of ethanol, acetate and biomass according to the following relationships: 1 mol mol ethanol^−1^, 1 mol mol acetate^−1^ and 0.1 mol C-mol biomass^−1^.

b The high standard deviation of these values was due to a single deviating measurement of ethanol in one experiment.

**Figure 3:**
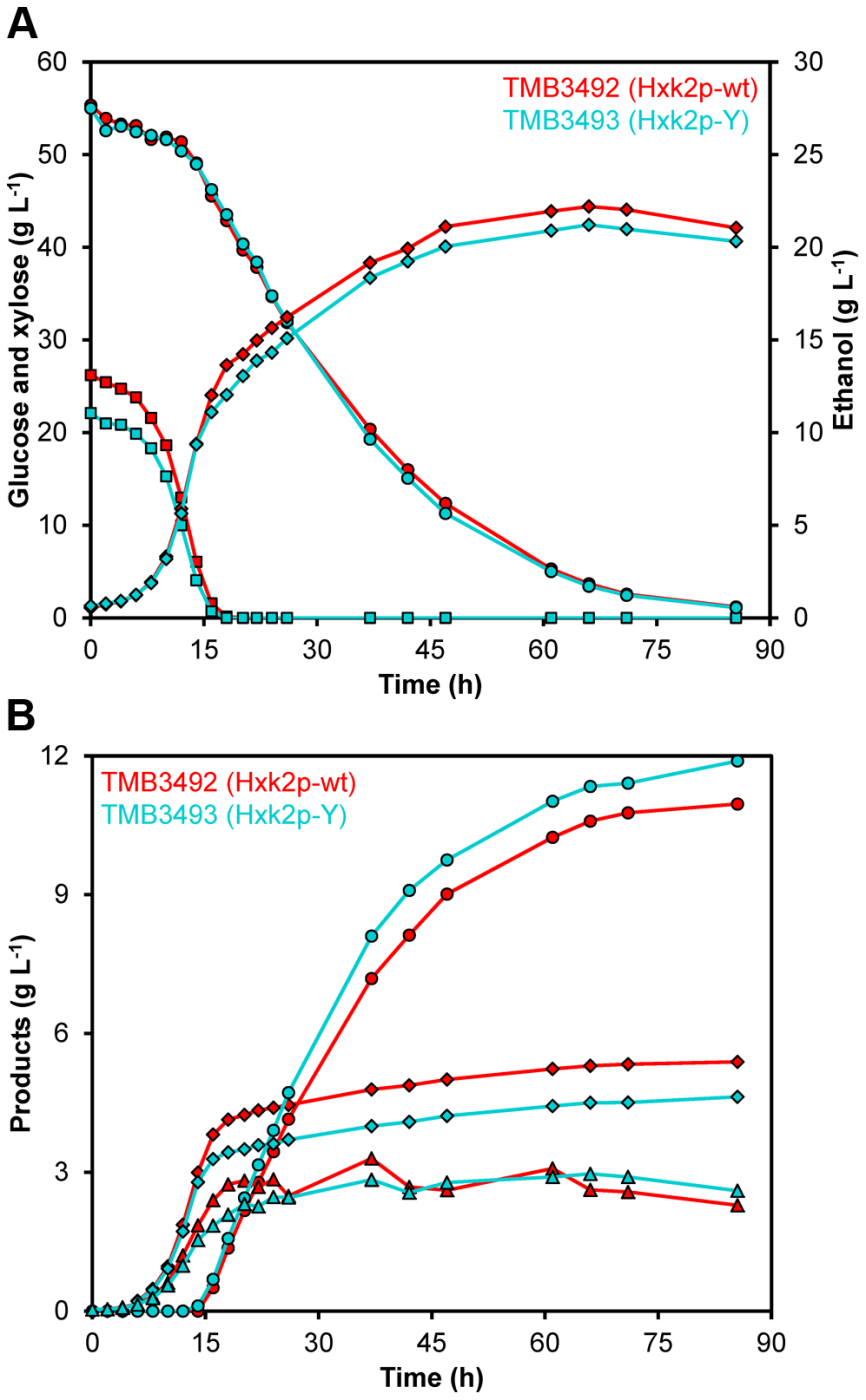
Anaerobic batch fermentation profiles of a glucose/xylose mixture. Fermentation experiments were performed using 2× YNB medium containing 20 g L^−1^ glucose and 50 g L^−1^ xylose. Figures show representative values from one experiment out of two biological duplicates using TMB3492 (Hxk2p-wt) (red) and TMB3493 (Hxk2p-Y) (turquoise). **A**) Fermentation profiles of glucose (▪) and xylose (•) consumption and production of ethanol (♦). **B**) Fermentation profiles of xylitol (•), glycerol (♦) and biomass (▴) formation.

To investigate whether the increased resistance toward xylose-induced inactivation affected batch fermentation of a glucose/xylose mixture, two new strains were constructed: TMB3492 (Hxk2p-wt) and TMB3493 (Hxk2p-Y). These strains assimilate xylose via the oxido-reductive pathway, consisting of a xylose reductase (XR) and a xylitol dehydrogenase (XDH) from *Scheffersomyces stipitis* and contain modifications known to improve xylose fermentation, such as increased activity of the enzymes in the non-oxidative pentose phosphate pathway and xylulokinase [45] as well as an engineered XR-variant with improved NADH preference [46]. The fermentation of 20 g L^−1^ glucose and 50 g L^−1^ xylose was evaluated in anaerobic batch cultivations (Fig. 3). However, there were no statistically significant differences between the two strains with regard to product yields per consumed xylose (Table S1) or per total amount of sugar consumed (Table S2). Strain TMB3493 had, however, a 64% faster (*P* = 0.095) maximum specific consumption rate of xylose compared to the control strain (Table S1). During the period of fastest xylose consumption, TMB3493 also had a maximum growth rate more than double that of the control strain (0.027 h^−1^ and 0.013 h^−1^, respectively) (Table S1). Although this difference was statistically significant at *P* = 0.037, the limited extension of this growth rate resulted in nearly identical overall productivities of the two strains (Table S2).

## Discussion

Previous studies on Hxk2p active-site have focused on the key catalytic residues involved in phosphate transfer, Ser158 or Asp211, by site-directed mutagenesis [22], [23]. In this study we describe the first condensed, rationally designed combinatorial library [41], [47] targeting the active-site in Hxk2p and the subsequent screening for mutations in the *HXK2* gene beneficial for resisting xylose-induced inactivation. This resulted in the identification of a protein variant, with the single amino acid substitution Phe159Tyr, which provides an increased glucose-phosphorylating activity in the presence of xylose.

**Figure 4:**
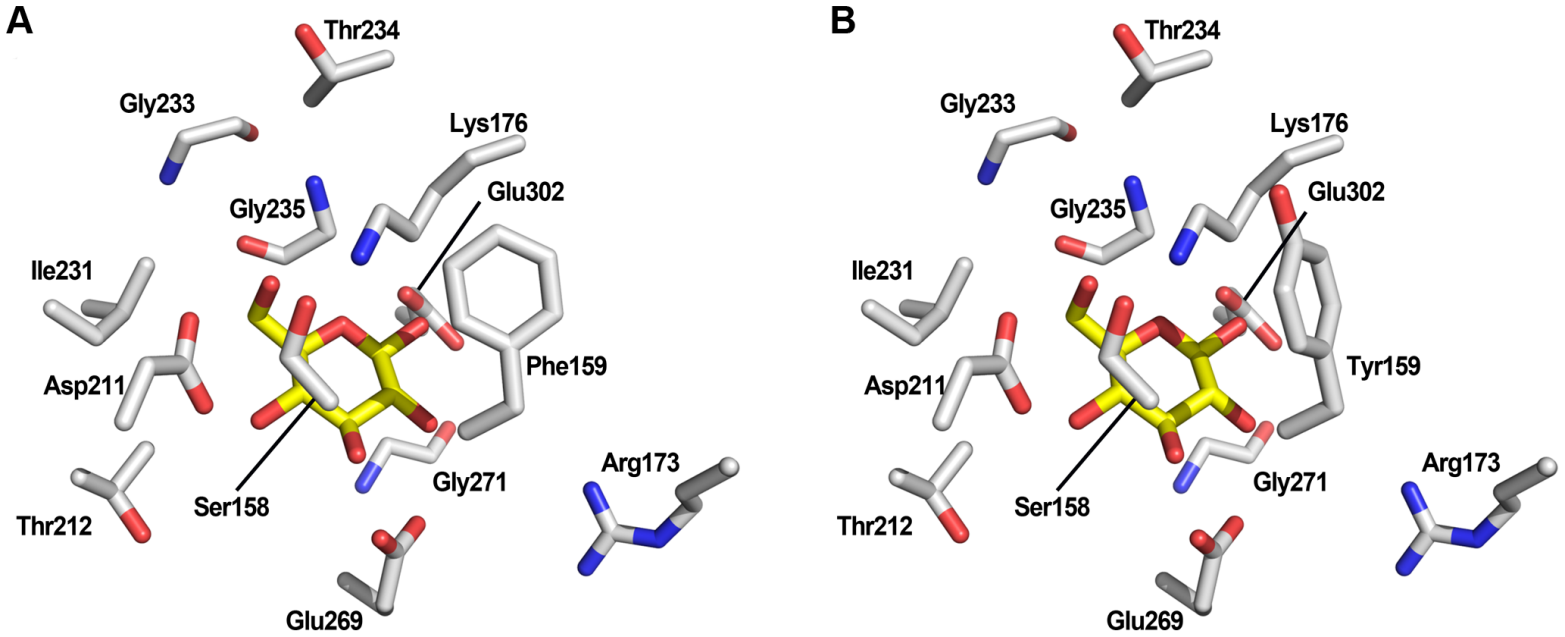
Homology models of the Hxk2p active site. The key residues in the active site of Hxk2p that selected for mutagenesis are displayed with corresponding label (see also Table 2). The position and orientation of the Phe159 and Tyr159 residues are shown in **A**) and **B**), respectively. Both figures show the orientation of the β-d-glucopyranose structure (yellow colour) in the active site.

The Phe159Tyr substitution does not impact negatively on the normal catalytic activity (Fig. 2), which is consistent with a widespread occurrence of tyrosine in position 159 among several hexokinases. For example, the Tyr159 residue is found in *S. cerevisiae* Hxk1p (PDB: 3B8A_X) and also in Hxk1p from the xylose-utilizing *Sc. stipitis* (NCBI RefSeq: XP_001386689). The occurrence of Tyr159 in the only hexokinase enzyme present in the xylose-utilizing *Sc. stipitis* supports our finding that it is beneficial for resisting xylose-induced inactivation. The Tyr159 residue is pointing towards the ATP-binding pocket and the additional hydroxyl group on the aromatic ring can generate a more polar environment at the edge of the glucose-binding pocket (Fig. 4). It is conceivable that the increased polarity generates an electron-rich draw on the phosphate group in ATP during the transition state, resulting in a lowered risk of autophosphorylation of the spatially close Ser158. Further studies are, however, needed to quantitate to what extent this novel substitution impacts i) the potential for autophosphorylation, ii) the kinetics of the enzyme and iii) the regulatory function.

The screening method used for identifying the Hxk2p-Y variant was designed to be rapid but still allowing the cells to grow at maximum growth rate under the given conditions. A short cultivation time reduces the extent of unknown adaptive modifications [48] and biofilm formation, which could lead to an inefficient selection for fast-growing strains as cells attached to surfaces and cells suspended in the broth would compete for the available nutrients [49]. There is, however, a significant drawback with the adopted method, i.e. the loss of selection pressure. The decrease in cell concentration during the washout reduced the capacity to consume glucose. Hence, the residual glucose inevitably accumulated over time, preventing xylose from entering the cells and act on the Hxk2p enzyme. To avoid this problem, screening should be performed in a turbidostat [49], [50] which can operate close to the maximum growth rate of the population while maintaining a low residual glucose concentration and high selection pressure.

The significant differences observed between strains TMB3466 and TMB3467 in xylose conversion at steady state (Table 3) suggest that Hxk2p has a role in the regulation of the endogenous xylose pathway in *S. cerevisiae*. There are four genes in *S. cerevisiae* encoding xylose reductases: *YJR096W*, *GCY1*, *GRE3* and *YPR1* [44]. Since the *GRE3* gene is disrupted in the parental strain TMB3042 used in this study, the observed conversion of xylose to xylitol is most likely catalysed by the *GCY1* or *YPR1* gene products. The BioGRID database (http://thebiogrid.org) lists one study in which a negative genetic interaction between *HXK2* and *GCY1* has been identified [51]. Hxk2p is known to regulate the expression of genes required for utilization of alternative carbon sources such as sucrose and galactose through the physical interactions with Med8p (*SUC2*) [15] and Rpt5p (*GAL1-10*) [52]. If xylose is also considered an alternative carbon source by *S. cerevisiae* it is plausible that the genes required for its utilization are also regulated by Hxk2p. However, further information is needed to confirm this hypothesis.

The new Hxk2p-Y variant can be applied in strains producing ethanol from lignocellulosic feedstocks. The hydrolysates produced from some of those materials are often rich in xylose [53]. Our hypothesis was that expressing a variant with improved repressive capability would increase the ethanol productivity by increasing the sugar consumption rate. This hypothesis is based on experimental results showing that Hxk2p acts as an activator of *HXT1*-expression and as a repressor of *HXT2* and *HXT4-7* [18], [19]. Hxt1p is a sugar transporter with low affinity and high transport capacity of both glucose and xylose; Hxt4p-7p, on the other hand, are high-affinity, low-capacity transporters [43]. When xylose is the sole carbon source mainly the low capacity transporters are present, even at high concentrations [28], [29], [30]. This makes the transport process inefficient since the available transporters easily become saturated with xylose. A fully functional Hxk2p in the presence of xylose might be enough to shift the expression of genes toward high-capacity transporters, leading to an increased uptake rate at high concentrations. The results presented here from anaerobic batch fermentations of glucose and xylose did indeed show a faster xylose uptake rate by the strain with the Hxk2p-Y variant (Table S1) which is in good agreement with the hypothesis. The increased uptake rate did, however, not lead to an increased production of ethanol but rather seemed to enable a faster initial growth rate on xylose. Unfortunately the extent of this growth period was very short indicating that the improved transport capacity must be combined with other regulatory factors induced by glucose. The identification of these factors requires further investigations. Still, the result obtained in this study is a first, but significant, step towards establishing a xylose repression signal in *S. cerevisiae*. Such a signal is most likely a prerequisite for an efficient conversion of xylose to bioethanol using recombinant *S. cerevisiae*.

## Supporting Information

Figure S1 **Construction of the mutated HXK2 megaprimer.** The figure outlines the OE-PCR-based strategy used to construct the mutated *HXK2* megaprimer. The reader is referred to the Supporting information for detailed information.
(TIFF)

Figure S2 **Sequencing of the HXK2-library.** The nucleotide sequences of the mutated regions are shown to the left. The top sequence is the native sequence and the bottom sequence contains the introduced degeneracy. Codons that were modified are underlined and the native and alternative amino acid residues are indicated with bold and white-on-black letters, respectively. The right panel shows the corresponding region from the electropherogram (note that Region 6 is shown as the reverse complement). Arrows indicate the point of the degeneracy and the dual signals show that the mutations are indeed introduced in the megaprimer.
(TIFF)

Figure S3 **Verification results of integration and gene deletion.** A) Primers used with template from CEN.PK2-1C (positive control) and TMB3462. B) Amplification results of the set-up shown in A). C) Amplification of each gene using specific primers listed in Table S5. The reaction with template from TMB3462 contained primers for all genes.
(TIFF)

Figure S4 **Illustration of the 39 bases long linker used to create a multiple cloning site into the pUG6AUR plasmid.**
(TIFF)

Figure S5 **The pUG62AUR plasmid with a multiple cloning site consisting of the** ***Avr*** **II,** ***Sph*** **I,** ***Sma*** **I and** ***Kpn*** **I restriction sites.**
(TIFF)

Figure S6 **The pUG62AUR-HXK1USDS plasmid containing homologous regions flanking the** ***HXK1*** **gene.**
(TIFF)

Figure S7 **The glucose phosphorylating activity in strains TMB3462 (3Δ) and TMB3463 (4Δ) relative to the wild-type CEN.PK2-1C strain.**
(TIFF)

Table S1 **Maximum specific consumption rates, production rates and yields in anaerobic batch fermentation of 20 g L^−1^ glucose and 50 g L^−1^ xylose by TMB3492 (Hxk2p-wt) and TMB3493 (Hxk2p-Y).** Values are given as mean ± standard deviation of two independent experiments.
(DOC)

Table S2 **Overall yields and production rates in anaerobic batch fermentation of 20 g L^−1^ glucose and 50 g L^−1^ xylose by TMB3492 (Hxk2p-wt) and TMB3493 (Hxk2p-Y).** Values are given as mean ± standard deviation of two independent experiments.
(DOC)

Table S3 **Primers used to construct the mutated** ***HXK2*** **megaprimer.**
(DOC)

Table S4 **Primers used to construct deletion cassettes.**
(DOC)

Table S5 **Primers used to confirm correct integration and gene deletion.**
(DOC)

Table S6 **Primers used to amplify additional fragments upstream and downstream the** ***HXK1*** **gene.** Restriction sites are indicated in bold.
(DOC)

Table S7 **Primers used to confirm correct integration of pUG62AUR-HXK1USDS and** ***HXK1*** **-gene deletion.**
(DOC)

Methods S1 Engineering yeast hexokinase 2 for improved tolerance toward xyloseinduced inactivation.
(PDF)
