# Supplementary figures and images for: Engineering Yeast Hexokinase 2 for Improved Tolerance Toward Xylose-Induced Inactivation
Source: PLoS One. 2013 Sep 6;8(9):e75055. doi: 10.1371/journal.pone.0075055 (PMC3765440; doi:10.1371/journal.pone.0075055)

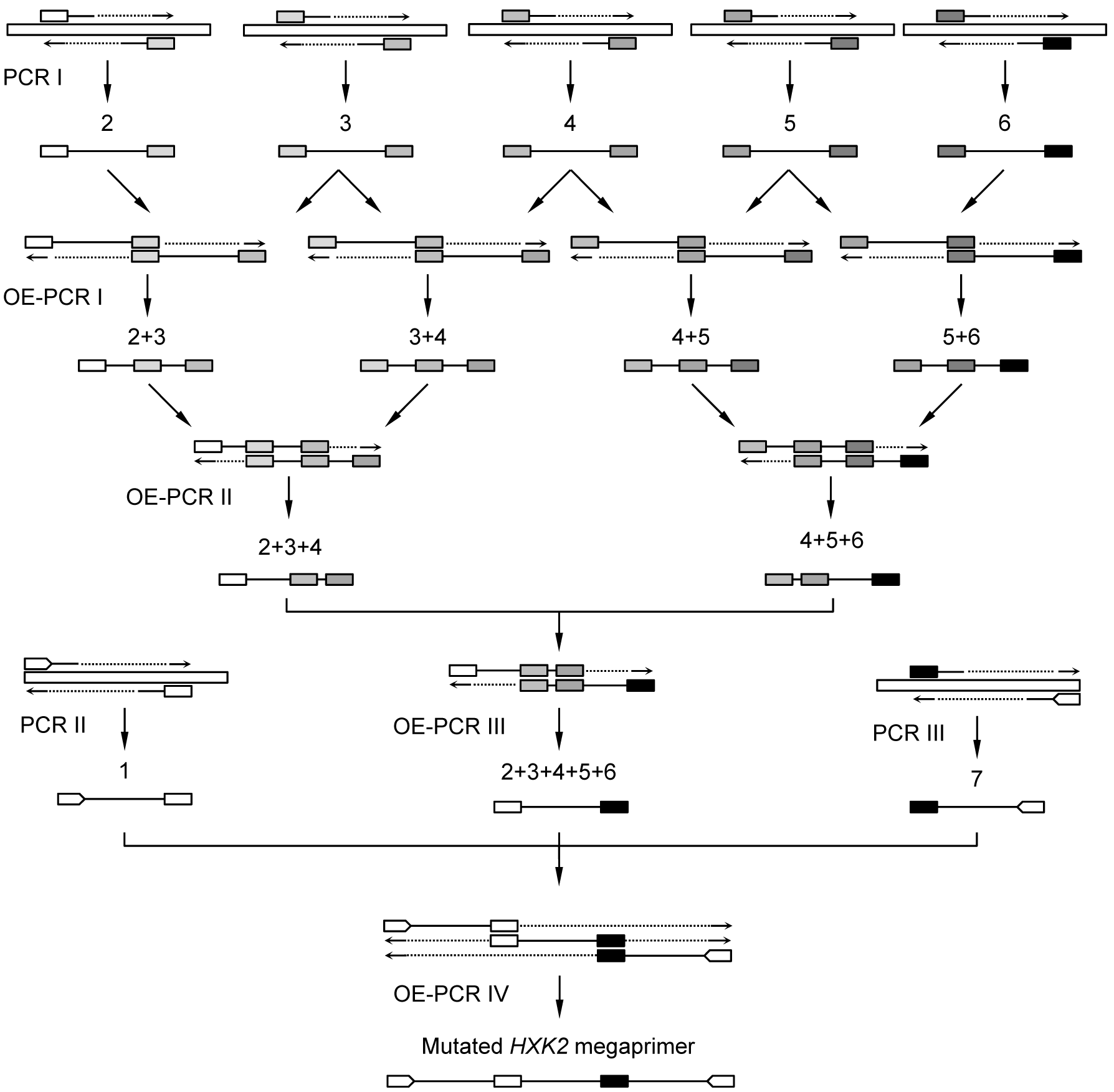

Supplement: Figure S1 — Construction of the mutated HXK2 megaprimer. The figure outlines the OE-PCR-based strategy used to construct the mutated HXK2 megaprimer. The reader is referred to the Supporting information for detailed information. (TIFF) [file pone.0075055.s001.tif]

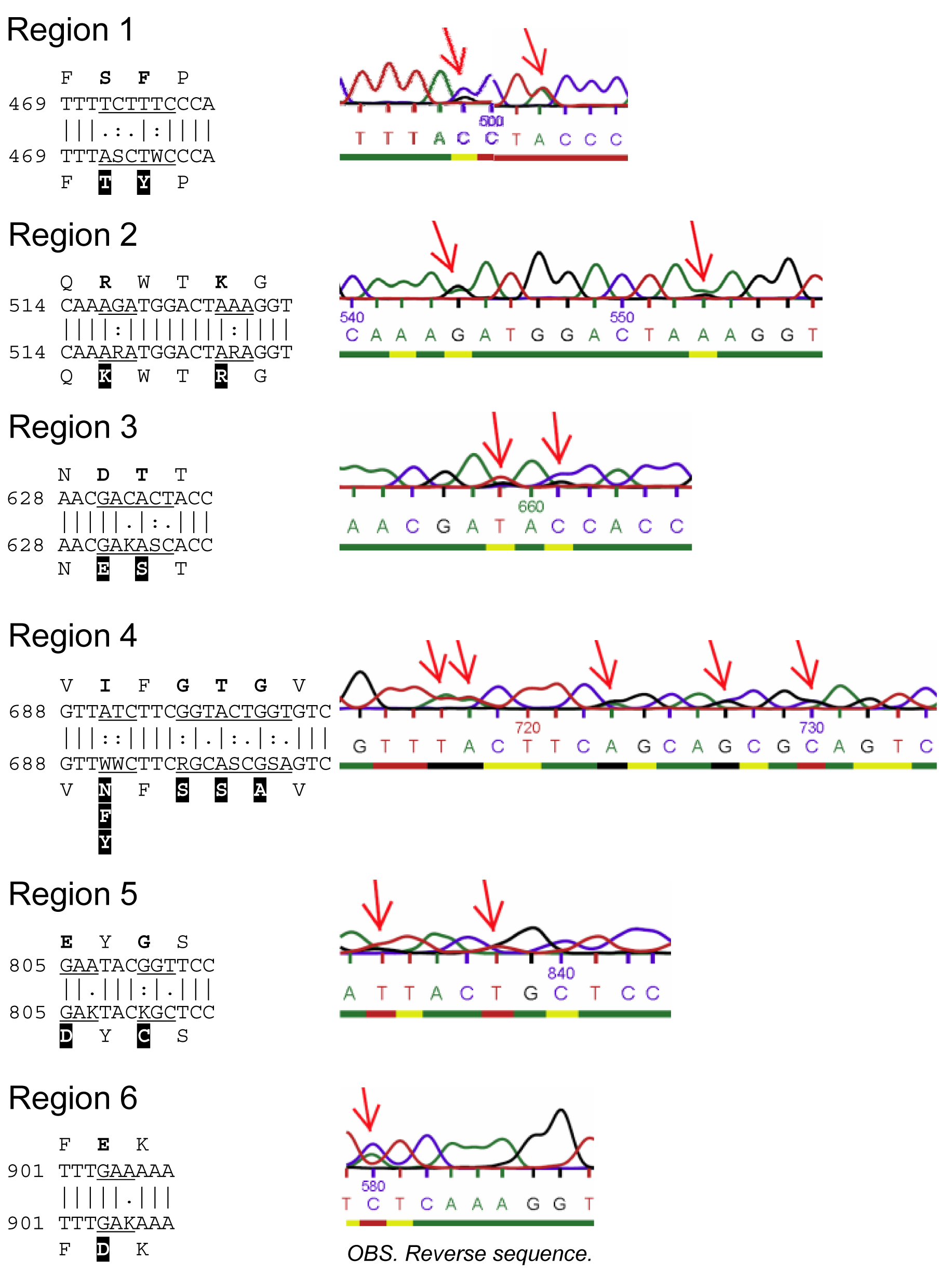

Supplement: Figure S2 — Sequencing of the HXK2-library. The nucleotide sequences of the mutated regions are shown to the left. The top sequence is the native sequence and the bottom sequence contains the introduced degeneracy. Codons that were modified are underlined and the native and alternative amino acid residues are indicated with bold and white-on-black letters, respectively. The right panel shows the corresponding region from the electropherogram (note that Region 6 is shown as the reverse complement). Arrows indicate the point of the degeneracy and the dual signals show that the mutations are indeed introduced in the megaprimer. (TIFF) [file pone.0075055.s002.tif]

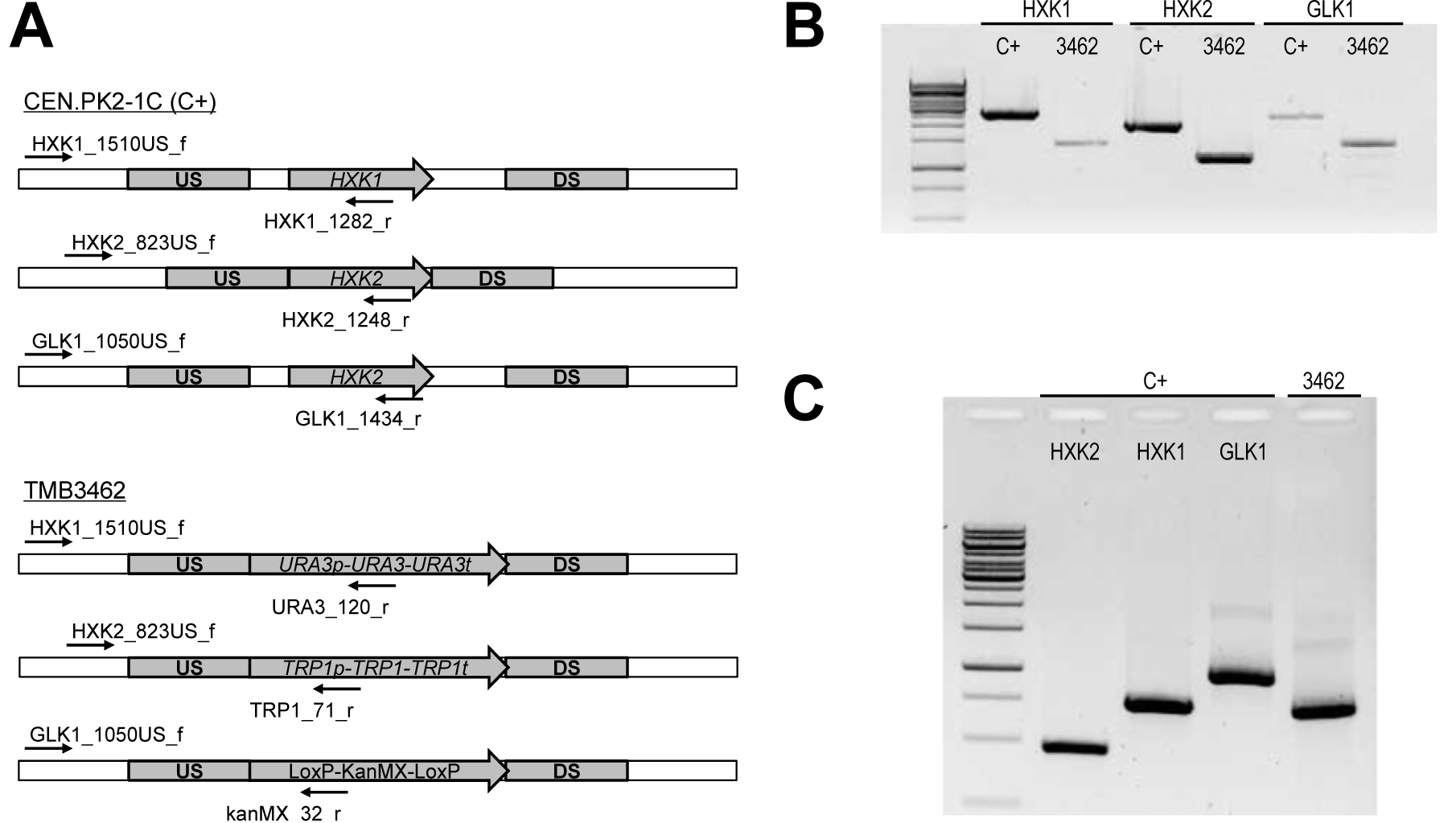

Supplement: Figure S3 — Verification results of integration and gene deletion. A) Primers used with template from CEN.PK2-1C (positive control) and TMB3462. B) Amplification results of the set-up shown in A). C) Amplification of each gene using specific primers listed in Table S5. The reaction with template from TMB3462 contained primers for all genes. (TIFF) [file pone.0075055.s003.tif]

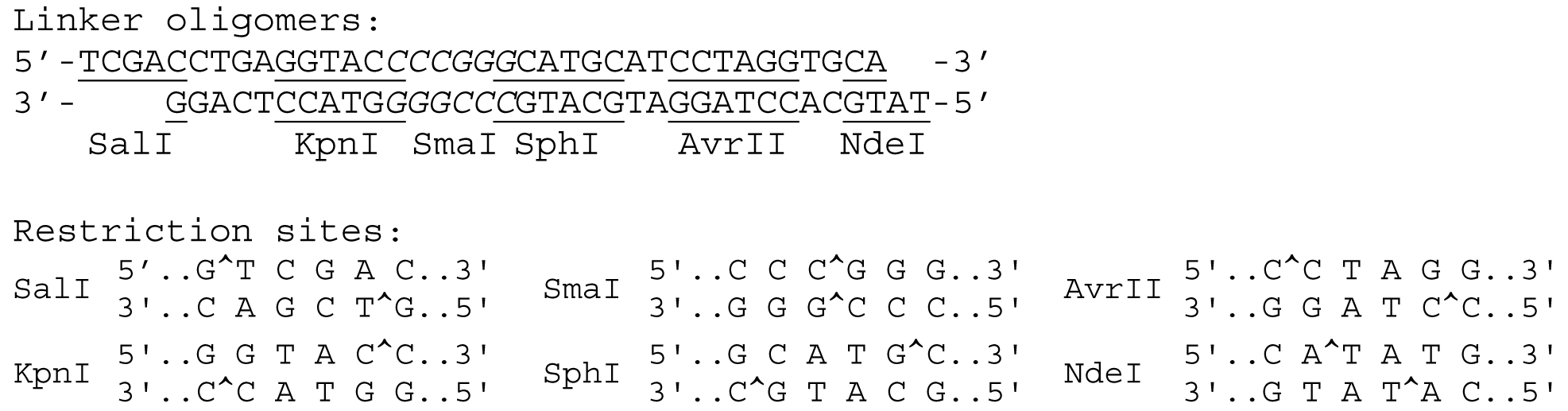

Supplement: Figure S4 — Illustration of the 39 bases long linker used to create a multiple cloning site into the pUG6AUR plasmid. (TIFF) [file pone.0075055.s004.tif]

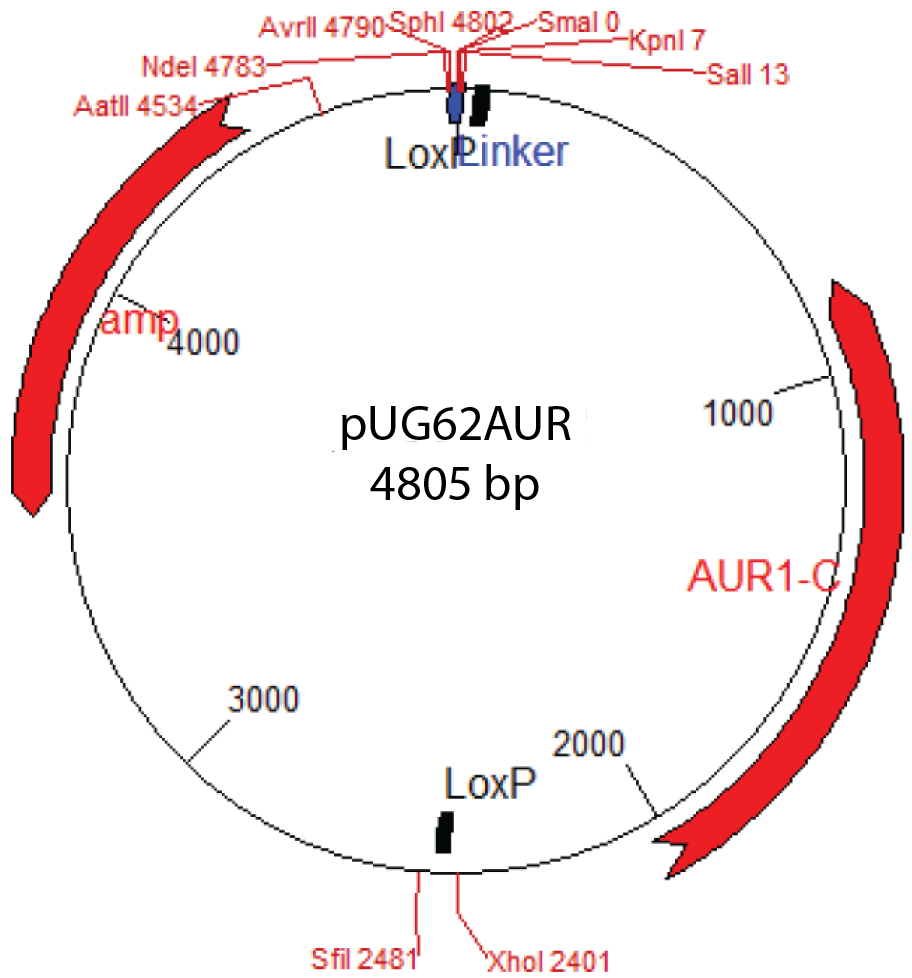

Supplement: Figure S5 — The pUG62AUR plasmid with a multiple cloning site consisting of the Avr II, Sph I, Sma I and Kpn I restriction sites. (TIFF) [file pone.0075055.s005.tif]

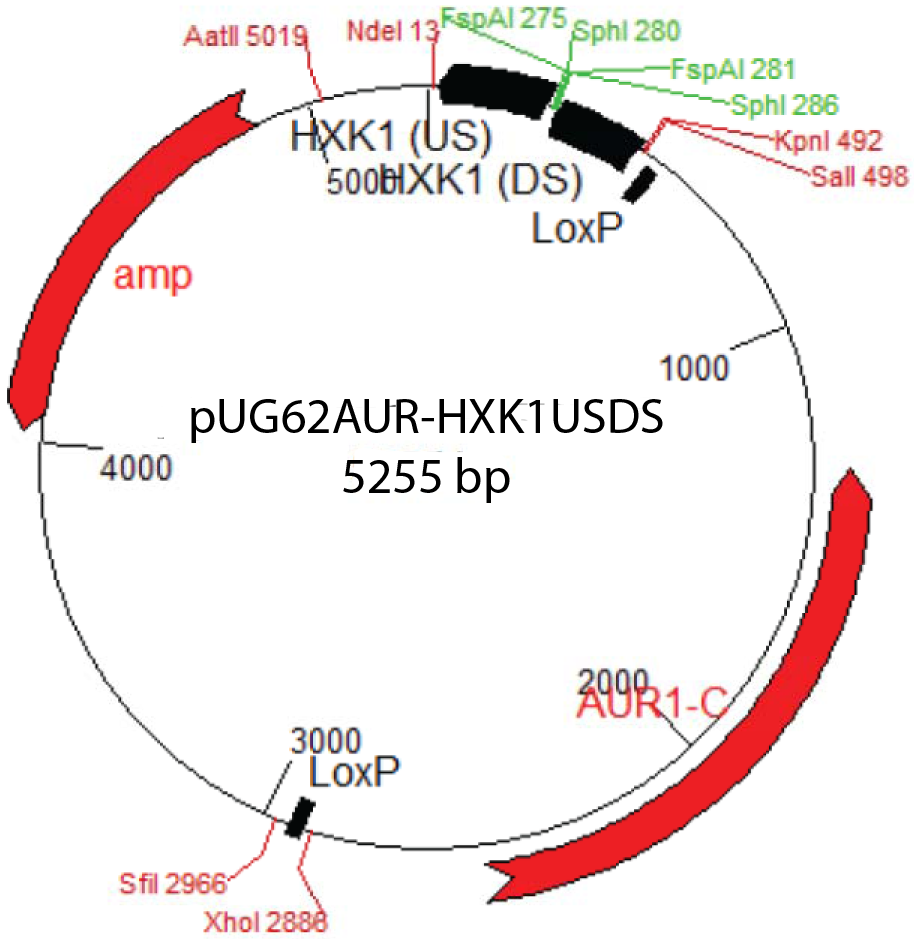

Supplement: Figure S6 — The pUG62AUR-HXK1USDS plasmid containing homologous regions flanking the HXK1 gene. (TIFF) [file pone.0075055.s006.tif]

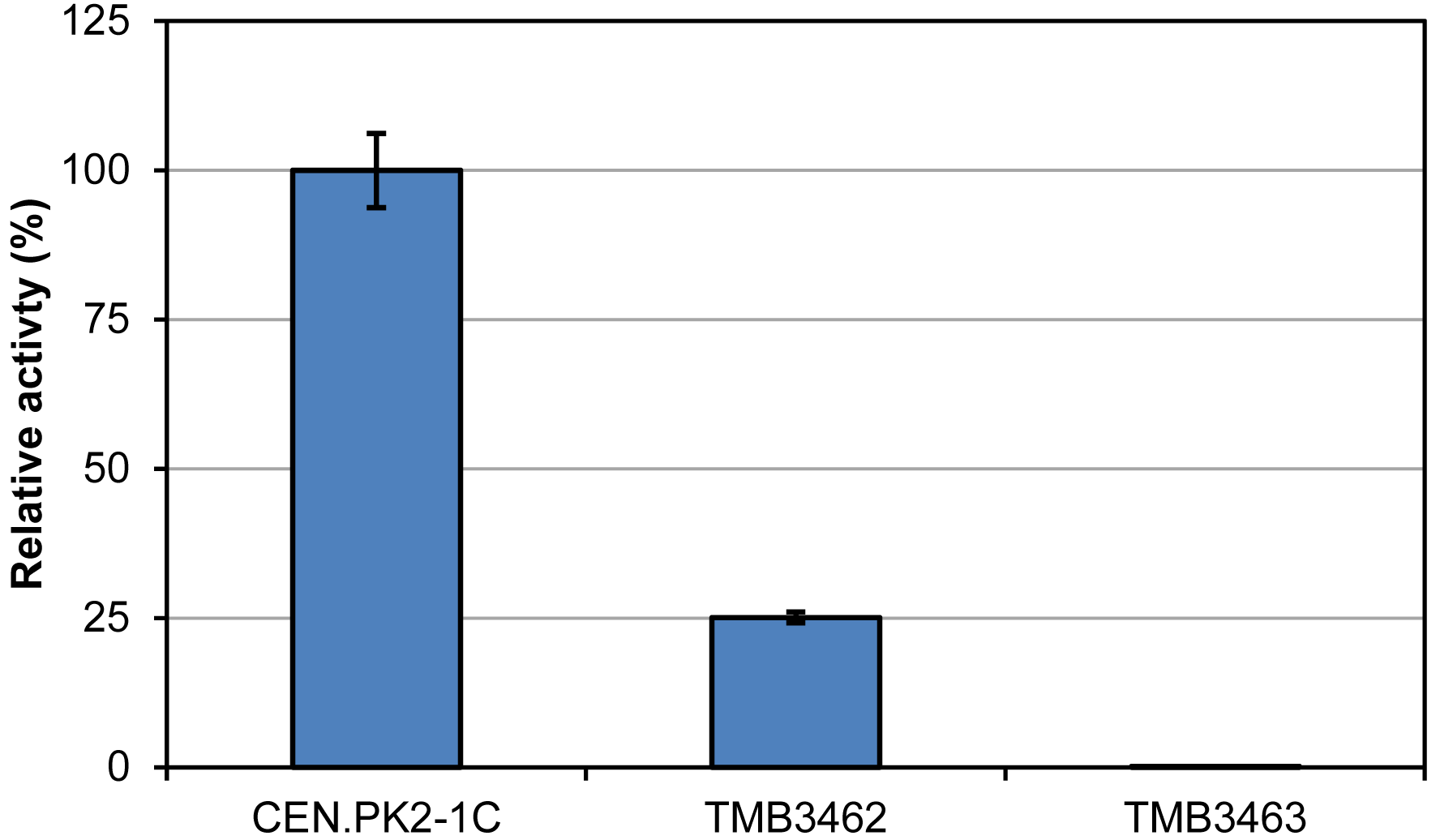

Supplement: Figure S7 — The glucose phosphorylating activity in strains TMB3462 (3Δ) and TMB3463 (4Δ) relative to the wild-type CEN.PK2-1C strain. (TIFF) [file pone.0075055.s007.tif]
