# Supplementary material for: Engineering Yeast Hexokinase 2 for Improved Tolerance Toward Xylose-Induced Inactivation
Source: PLoS One. 2013 Sep 6;8(9):e75055. doi: 10.1371/journal.pone.0075055 (PMC3765440; doi:10.1371/journal.pone.0075055)
Supplement: Table S1 — Maximum specific consumption rates, production rates and yields in anaerobic batch fermentation of 20 g L−1 glucose and 50 g L−1 xylose by TMB3492 (Hxk2p-wt) and TMB3493 (Hxk2p-Y). Values are given as mean ± standard deviation of two independent experiments. (DOC) [file pone.0075055.s008.doc]

Supporting Table S1. Maximum specific consumption rates, production rates and yields in anaerobic batch fermentation of 20 g L^-1^ glucose and 50 g L^-1^ xylose by TMB3492 (Hxk2p-wt) and TMB3493 (Hxk2p-Y).

Values are given as mean ± standard deviation of two independent experiments.

|  | Glucose phase | | | | Xylose phase | | | |
| --- | --- | --- | --- | --- | --- | --- | --- | --- |
|  | TMB3492 | TMB3493 | | TMB3492 | | TMB3493 | |  |
| *µ*_max_ (h^-1^) | 0.35±0.02 | | 0.34±0.01 | | 0.013±0.000 | | 0.027±0.004 | |
| Specific rates (mmol g CDW^-1^ h^-1^) | | | | | | | | |
| *r*_max,glc_ | -20.6±3.2 | | -21.8±0.4 | | – | | – | |
| *r*_max,xyl_ | – | | – | | -3.48±0.35 | | -5.72±0.99 | |
| *r*_max,xylt_ | – | | – | | 0.909±0.017 | | 0.960±0.014 | |
| *r*_max,glyc_ | 6.31±0.49 | | 6.61±0.42 | | 0.085±0.041 | | 0.052±0.037 | |
| *r*_max,ac_ | 1.04±0.02 | | 1.22±0.09 | | 0.040±0.021 | | 0.028±0.005 | |
| *r*_max,etoh_ | 39.1±5.6 | | 41.3±2.5 | | 1.60±0.40 | | 1.34±0.98 | |
| Yields (g g sugar^-1^) | | | | | | | | |
| *Y*_xylt/s_ | – | | – | | 0.261±0.007 | | 0.270±0.003 | |
| *Y*_glyc/s_ | 0.129±0.005 | | 0.127±0.007 | | 0.028±0.001 | | 0.027±0.000 | |
| *Y*_ac/s_ | 0.015±0.002 | | 0.015±0.000 | | 0.009±0.003 | | 0.008±0.002 | |
| *Y*_etoh/s_ | 0.398±0.012 | | 0.392±0.036 | | 0.251±0.003 | | 0.255±0.009 | |
| *Y*_X/s_ | 0.078±0.005 | | 0.070±0.002 | | 0.056±0.013 | | 0.055±0.008 | |
