# Supplementary material for: Engineering Yeast Hexokinase 2 for Improved Tolerance Toward Xylose-Induced Inactivation
Source: PLoS One. 2013 Sep 6;8(9):e75055. doi: 10.1371/journal.pone.0075055 (PMC3765440; doi:10.1371/journal.pone.0075055)
Supplement: Table S2 — Overall yields and production rates in anaerobic batch fermentation of 20 g L−1 glucose and 50 g L−1 xylose by TMB3492 (Hxk2p-wt) and TMB3493 (Hxk2p-Y). Values are given as mean ± standard deviation of two independent experiments. (DOC) [file pone.0075055.s009.doc]

Supporting Table S2. Overall yields and production rates in anaerobic batch fermentation of 20 g L^-1^ glucose and 50 g L^-1^ xylose by TMB3492 (Hxk2p-wt) and TMB3493 (Hxk2p-Y).

Values are given as mean ± standard deviation of two independent experiments.

| Overall yields (g g total sugar^-1^) | | | Overall production rates (g L^-1^ h^-1^) | | |
| --- | --- | --- | --- | --- | --- |
|  | TMB3492 | TMB3493 |  | TMB3492 | TMB3493 |
| *Y*_xylt/s_ | 0.144±0.010 | 0.158±0.002 | *q_xylt_* | 0.133±0.007 | 0.139±0.000 |
| *Y*_glyc/s_ | 0.061±0.008 | 0.059±0.002 | *q_glyc_* | 0.057±0.008 | 0.052±0.003 |
| *Y*_ac/s_ | 0.011±0.002 | 0.012±0.001 | *q_ac_* | 0.010±0.002 | 0.010±0.001 |
| *Y*_etoh/s_ | 0.258±0.004 | 0.262±0.004 | *q_etoh_* | 0.240±0.000 | 0.231±0.001 |
| *Y*_X/s_ | 0.032±0.005 | 0.036±0.003 | *q_X_* | 0.029±0.004 | 0.031±0.002 |
