# Supplementary material for: Engineering Yeast Hexokinase 2 for Improved Tolerance Toward Xylose-Induced Inactivation
Source: PLoS One. 2013 Sep 6;8(9):e75055. doi: 10.1371/journal.pone.0075055 (PMC3765440; doi:10.1371/journal.pone.0075055)
Supplement: Table S3 — Primers used to construct the mutated HXK2 megaprimer. (DOC) [file pone.0075055.s010.doc]

Supporting Table S3. Primers used to construct the mutated *HXK2* megaprimer.

| Name | | Sequence (with wobble positions in bold letters) |
| --- | --- | --- |
| Degenerate primers | | |
| Group_1_f | | 5’-CATTGGGTTTCACCTTTA**S**CT**W**CCCAGCTTCTCAAAAC-3’ |
| Group_1_r | | 5’-GTTTTGAGAAGCTGGG**W**AG**S**TAAAGGTGAAACCCAATG-3’ |
| Group_2_f | | 5’-GAAGGTATCTTGCAAA**R**ATGGACTA**R**AGGTTTTGATATTCC-3’ |
| Group_2_r | | 5’-GGAATATCAAAACCT**Y**TAGTCCAT**Y**TTTGCAAGATACCTTC-3’ |
| Group_3_f | | 5’-GTTGCTTTGATAAACGA**K**A**S**CACCGGTACTTTGGTTG-3’ |
| Group_3_r | | 5’-CAACCAAAGTACCGGTG**S**T**M**TCGTTTATCAAAGCAAC-3’ |
| Group_4_f | | 5’-GAAACTAAGATGGGTGTT**WW**CTTC**R**GCA**S**CG**S**AGTCAATGGTGCTTAC-3’ |
| Group_4_r | | 5’-GTAAGCACCATTGACT**S**CG**S**TGC**Y**GAAG**WW**AACACCCATCTTAGTTTC-3’ |
| Group_5_f | | 5’-CAATGGCCATCAACTGTGA**K**TAC**K**GCTCCTTCGATAATGAAC-3’ |
| Group_5_r | | 5’-GTTCATTATCGAAGGAGC**M**GTA**M**TCACAGTTGATGGCCATTG-3’ |
| Group_6_f | | 5’-CAGGCCAACAAACCTTTGA**K**AAAATGTCTTCTGGTTAC-3’ |
| Group_6_r | | 5’-GTAACCAGAAGACATTTT**M**TCAAAGGTTTGTTGGCCTG-3’ |
| Additional primers | | |
| Yip128-F1 | 5’-GGCCTTTTGCTGGCCTTTTG-3’ | |
| Yip128-R1 | 5’-AAGGGGGATGTGCTGCAAGG-3’ | |
