# Supplementary material for: Engineering Yeast Hexokinase 2 for Improved Tolerance Toward Xylose-Induced Inactivation
Source: PLoS One. 2013 Sep 6;8(9):e75055. doi: 10.1371/journal.pone.0075055 (PMC3765440; doi:10.1371/journal.pone.0075055)
Supplement: Table S4 — Primers used to construct deletion cassettes. (DOC) [file pone.0075055.s011.doc]

Supporting Table S4. Primers used to construct deletion cassettes.

| Name | Sequence |
| --- | --- |
| HXK2_US_f | 5’-GGTACCTAGAAATGGCTATCATGC-3’ |
| HXK2_US_r | 5’-TATATATAGTAATGTCGTTTATTTAATTAGCGTACTTATTATGTGTGG-3’ |
| HXK2_DS_f | 5’-GATAGGGTTGAGTGTTGTTACTTAATTTGTAAATTAAGTTTGAACAACAAG-3’ |
| HXK2_DS_r | 5’-AGAAGAATCCACGCGTAAAAATCG-3’ |
| TRP1_f | 5’-TAAGTACGCTAATTAAATAAACGACATTACTATATATATAATATAGGAAG-3’ |
| TRP1_r | 5’-CTTAATTTACAAATTAAGTAACAACACTCAACCCTATCTAGGTC-3’ |
| HXK1_US_f | 5’-TGGCGTGGGGTGGGGTGATT-3’ |
| HXK1_US_r | 5’-GTTAAGCCAGCCCCGACACCGGGCACGTGCGGGAGTTT-3’ |
| HXK1_DS_f | 5’-GTCTATCAGGGCGATGGCCCGCGACCCAGATTGCAGAAGATCCC-3’ |
| HXK1_DS_r | 5’-AGGTGCCCTTGCTAGCAT-3’ |
| URA3_f | 5’-AAACTCCCGCACGTGTGCCCGGTGTCGGGGCTGGCTTAAC-3’ |
| URA3_r | 5’-GGGATCTTCTGCAATCTGGGTCGCGGGCCATCGCCCTGATAGAC-3’ |
| GLK1_US_f | 5’-AGAGGAGGCGAGCAGCAGGG-3’ |
| GLK1_US_r | 5’-GTCGACCTGCAGCGTACGAAAGTGCCCACCGTTTGAGCGT-3’ |
| GLK1_DS_f | 5’-ATCAGATCCACTAGTGGCCTATGCGACAGCCTCGCCCTCTTCCGT-3’ |
| GLK1_DS_r | 5’-CGGGCAGTGCAGTGTGAGGG-3’ |
| loxPKanMX_f | 5’-ACGCTCAAACGGTGGGCACTTTCGTACGCTGCAGGTCGAC-3’ |
| loxPKanMX_R | 5’-ACGGAAGAGGGCGAGGCTGTCGCATAGGCCACTACCTCTTCCGT-3’ |
