# Supplementary material for: Engineering Yeast Hexokinase 2 for Improved Tolerance Toward Xylose-Induced Inactivation
Source: PLoS One. 2013 Sep 6;8(9):e75055. doi: 10.1371/journal.pone.0075055 (PMC3765440; doi:10.1371/journal.pone.0075055)
Supplement: Table S5 — Primers used to confirm correct integration and gene deletion. (DOC) [file pone.0075055.s012.doc]

Supporting Table S5. Primers used to confirm correct integration and gene deletion.

| Name | Sequence | Application |
| --- | --- | --- |
| HXK2_823US_f | 5’-ACCACACGCATGCCTTCATTCC-3’ | Chromosomal integration |
| TRP1_71_r | 5’-GCGGCCTCTGTGCTCTGCAA-3’ |
| HXK2_773_f | 5’-TTCCACCATCTGCTCCAATGGC-3’ | Gene deletion |
| HXK2_1248_r | 5’-TGCAGCAATGTGACCGGTCTTG-3’ |
| HXK1_1510US_f | 5’-AGCGGTTCGCTTCCAGCACC-3’ | Chromosomal integration |
| URA3_120_r | 5’-GGTGGTACGAACATCCAATGAAGCA-3’ |
| HXK1_547_f | 5’-GTCGAAGGCCACGATGTCGTCC-3’ | Gene deletion |
| HXK1_1282_r | 5’-CCCTTAGCGGCGGCTTCCTT-3’ |
| GLK1_1050US_f | 5’-ACGGCGACAGCCGGTTGGCTT-3’ | Chromosomal integration |
| kanMX_32_r | 5’-CGCGGCCTCGAAACGTGAGT-3’ |
| GLK1_433_f | 5’-CCGGACGAGTTGGCCAAGGG-3’ | Gene deletion |
| GLK1_1434_r | 5’-CCTCTCACCCTCGGCACCCA-3’ |
