# Supplementary material for: Engineering Yeast Hexokinase 2 for Improved Tolerance Toward Xylose-Induced Inactivation
Source: PLoS One. 2013 Sep 6;8(9):e75055. doi: 10.1371/journal.pone.0075055 (PMC3765440; doi:10.1371/journal.pone.0075055)
Supplement: Table S6 — Primers used to amplify additional fragments upstream and downstream the HXK1 gene. Restriction sites are indicated in bold. (DOC) [file pone.0075055.s013.doc]

Supporting Table S6. Primers used to amplify additional fragments upstream and downstream the *HXK1* gene.

Restriction sites are indicated in bold.

| Name | Sequence | Restriction site |
| --- | --- | --- |
| HXK1_US2_f | 5’-TAG**GCATGC**GCATTGGTACCTTAGGACCGTTGAG-3’ | *Sph*I |
| HXK1_US2_r | 5’-CG**CCTAGG**GATTGAGTTGTTTGGGTGAGTTTG-3’ | *Avr*II |
| HXK1_DS2_f | 5’-ACT**GGTACC**TTGGTCTTCTTCATGCATCATTTCA-3’ | *Kpn*I |
| HXK1_DS2_r | 5’-TTG**GCATGC**ATCAGCTATAAGAGACGAAATTGCT-3’ | *Sph*I |
