# Supplementary material for: Engineering Yeast Hexokinase 2 for Improved Tolerance Toward Xylose-Induced Inactivation
Source: PLoS One. 2013 Sep 6;8(9):e75055. doi: 10.1371/journal.pone.0075055 (PMC3765440; doi:10.1371/journal.pone.0075055)
Supplement: Table S7 — Primers used to confirm correct integration of pUG62AUR-HXK1USDS and HXK1 -gene deletion. (DOC) [file pone.0075055.s014.doc]

Supporting Table S7. Primers used to confirm correct integration of pUG62AUR-HXK1USDS and *HXK1*-gene deletion.

| Name | Sequence | Application |
| --- | --- | --- |
| HXK1_1510US_f | 5’-AGCGGTTCGCTTCCAGCACC-3’ | Chromosomal integration |
| AmpR-R130 | 5’-AATGATACCGCGAGACCCAC-3’ |
| HXK1_547_f | 5’-GTCGAAGGCCACGATGTCGTCC-3’ | Gene deletion |
| HXK1_1282_r | 5’-CCCTTAGCGGCGGCTTCCTT-3’ |
