# Supplementary material for: Engineering Yeast Hexokinase 2 for Improved Tolerance Toward Xylose-Induced Inactivation
Source: PLoS One. 2013 Sep 6;8(9):e75055. doi: 10.1371/journal.pone.0075055 (PMC3765440; doi:10.1371/journal.pone.0075055)
Supplement: Methods S1 — Engineering yeast hexokinase 2 for improved tolerance toward xyloseinduced inactivation. (PDF) [file pone.0075055.s015.pdf]

Supporting information

Engineering yeast hexokinase 2 for improved tolerance toward xylose-induced inactivation

Basti Bergdahl[1,*], Anders G. Sandström[1], Celina Borgström[1], Tarinee Boonyawan[1,†], Ed W.J. van Niel[1] and Marie F. Gorwa-Grauslund[1]

[1] Division of Applied Microbiology, Department of Chemistry, Lund University, P.O. Box 124, SE-22100 Lund, Sweden

[†] Current address: Centre of Medical Excellence, Faculty of Medicine, Chiang Mai University, 110 Intavaroros, Amphur Muang, Chiang Mai 50200, Thailand.

* To whom correspondence should be addressed. E-mail: basti.bergdahl@tmb.lth.se (B.B.)

Contents

Supporting Methods and Figures **2**

Construction of the E. coli library of Hxk2p-variants 2

Cloning of the *HXK2* locus 2

Generating the plasmid library using the MEGAWHOP procedure 4

Construction of strains TMB3460, TMB3461 and TMB3462 6

Construction of TMB3460 (*hxk2*-Δ) 6

Construction of TMB3461 (*hxk2*-Δ *hxk1*-Δ*1*) 6

Construction of TMB3462 (*hxk2*-Δ *hxk1*-Δ*1 glk1*-Δ) 7

Construction of the screening strain TMB3463 8

Generation of plasmid pUG62AUR 8

Construction of TMB3463 (*hxk2*-Δ *hxk1*-Δ*1 glk1*-Δ *hxk1*-Δ*2*) 9

Supporting Tables **12**

Supporting Methods and Figures

Construction of the *E. coli* library of Hxk2p-variants

Cloning of the *HXK2* locus

The locus was amplified using primers HXK2_loc_f (5'-GCT TGC ATG CAC GCC ATA GAA GAG CAA TTT CCG TCC-3') and HXK2_loc_r (5'-CCG GGG ATC CGA GAG GGT TAA AAT TGG CGT GCA ATT TTA TGA AG-3'). The high fidelity Phusion Hotstart II polymerase (Thermo Scientific, USA) was used with the following PCR program: 30 s initial denaturation at 98°C, 30 cycles of 10 s denaturation at 98°C, 30 s annealing at 65°C and 1 min elongation at 72°C, and a final 10 min elongation step at 72°C. The resulting DNA fragment was digested with *Bam*HI and *Sph*I (FastDigest, Thermo Scientific, USA) at 37°C for 30 min and ligated into YIplac128, linearized with the same restriction enzymes. The ligation system was subsequently used to transform *E. coli* NEB5α.

Construction of the mutated *HXK2* megaprimer

The strategy used to construct the mutated *HXK2* megaprimer is outlined in Figure S1 and the primers are listed in Table S3.

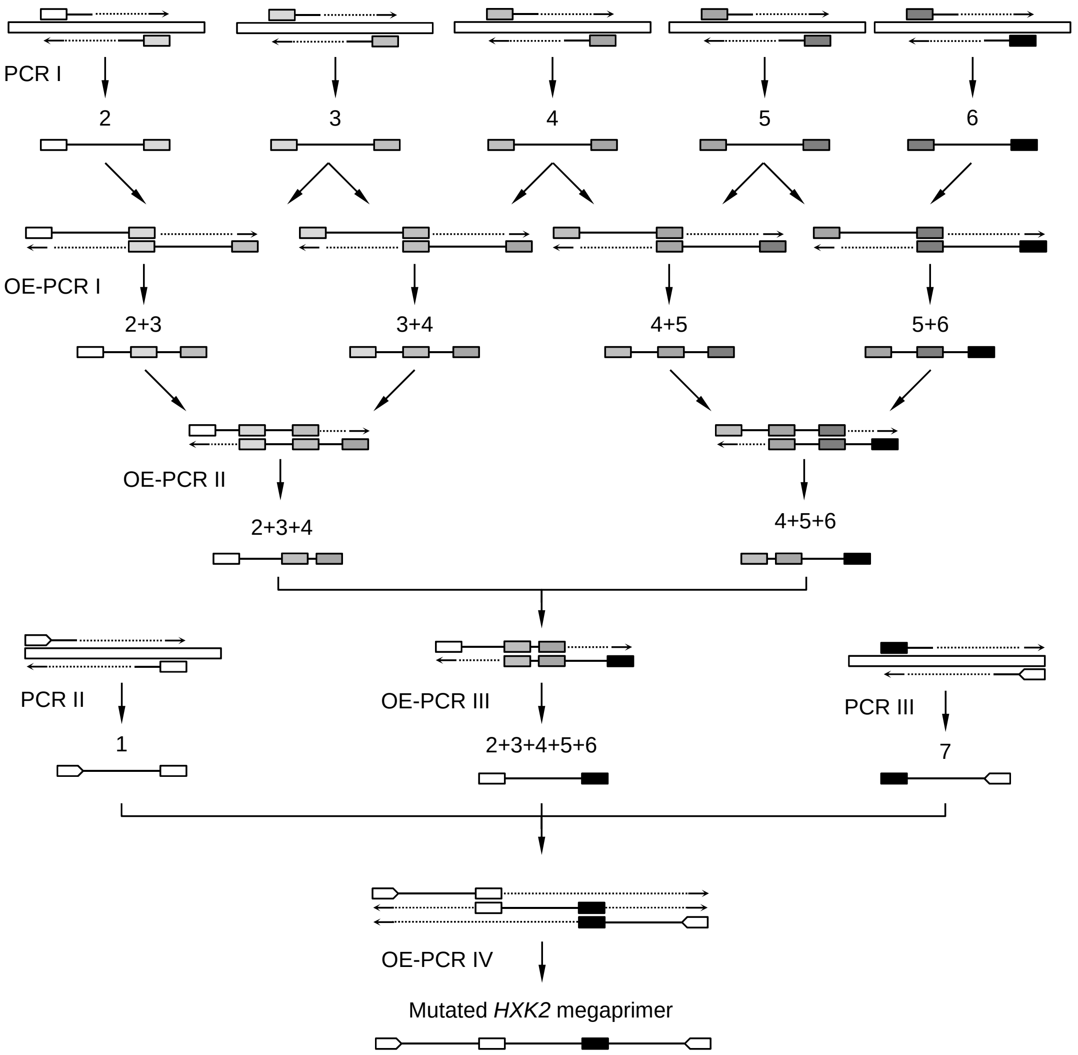

Supporting Figure S1. Construction of the mutated *HXK2* megaprimer

Unless stated otherwise the PCR reactions contained 1× HF Buffer, 0.2 mM of each dNTP, 0.01 U μL^{-1} Phusion Hotstart DNA Polymerase II and 0.5 μM each of forward and reverse primers. The reaction volume was 50 μL. OE-PCR reactions contained 30 fmol of each fragment. DNA fragments amplified by PCR were purified using GeneJet PCR Purification Kit (Thermo Scientific, USA) unless stated otherwise.

PCR I: Mutated fragments were amplified from YIpBB5 using the following PCR program: 30 s denaturation at 98°C, 30 cycles of 10 s denaturation at 98°C, 5 s annealing and extension at 72°C and 1 cycle of 10 min extension at 72°C. Primers were used according to the following scheme:
Fragment 2: Group_1_f and Group_2_r
Fragment 3: Group_2_f and Group_3_r
Fragment 4: Group_3_f and Group_4_r
Fragment 5: Group_4_f and Group_5_r
Fragment 6: Group_5_f and Group_6_r

PCR II: The non-mutated fragments were amplified from YIpBB5 using the following PCR program: 30 s denaturation at 98°C, 20 cycles of 10 s denaturation at 98°C, 15 s annealing at 72°C (-0.5°C $cycle^{-1}$), 30 s extension at 72°C, 16 cycles of 10 s denaturation at 98°C, 15 s annealing at 62.4°C, 30 s extension at 72°C and 1 cycle of 10 min extension at 72°C. Primers were used according to the following scheme:
Fragment 1: Yip128-F1 and Group_1_r
Fragment 7: Group_6_f and Yip128-R1

OE-PCR I: The two fragments were joined using 1× GC buffer in the reaction mix and the following PCR program: 30 s denaturation at 98°C, 16 cycles of 10 s denaturation at 98°C, 30 s annealing at 70°C (-0.5°C $cycle^{-1}$) and 30 s extension at 72°C. At this point the reaction was halted at 4°C until primers had been added after which the program was repeated. The program was finished with a final 10 min extension at 72°C. The primers were added to a final concentration of 0.52 μM according to the following scheme:
Fragment 2+3: Group_1_f and Group_3_r
Fragment 3+4: Group_2_f and Group_4_r
Fragment 4+5: Group_3_f and Group_5_r
Fragment 5+6: Group_4_f and Group_6_r

OE-PCR II: The two fragments were joined using the same procedure as in OE-PCR I. The primers were added to a final concentration of 0.52 μM according to the following scheme:
Fragment 2+3+4: Group_1_f and Group_4_r
Fragment 4+5+6: Group_3_f and Group_6_r

OE-PCR III: The two fragments were joined using the same procedure as in OE-PCR I. The primers Group_1_f Group_6_r and were added to a final concentration of 0.52 μM.

OE-PCR IV: The three fragments were joined using the following PCR program: 30 s denaturation at 98°C, 16 cycles of 10 s denaturation at 98°C, 30 s annealing at 70°C (-0.5°C $cycle^{-1}$), 30 s extension at 72°C and 1 cycle of 5 min extension at 72°C. At this point the reaction was halted at 4°C until primers had been added after which the program was repeated. The program was finished with a final 10 min extension at 72°C. The primers Yip128-F1 and Yip128-R1 were added to a final concentration of 0.52 μM.

The final megaprimer was purified from 0.8% agarose gel using the QIAquick Gel Extraction Kit (Qiagen, Germany).

Generating the plasmid library using the MEGAWHOP procedure

The MEGAWHOP reaction mixture (50 µL) contained the following: 1× HF Buffer, 0.2 mM of each dNTP, 503 ng of HXK2 megaprimer, 50.1 ng of template plasmid YIpBB5 and 0.02 U μL^{-1} Phusion Hotstart DNA Polymerase II. The whole plasmid amplification was performed using the following PCR program: 30 s denaturation at 98°C, 25 cycles of 10 s denaturation at 98°C, 15 s annealing at 58°C and 3 min extension at 72°C.

After completing the PCR reaction, 0.8 µL *Dpn*I (20 U μL^{-1}) was added to 40 µL of the MEGAWHOP reaction mixture and incubated for 2 h at 37°C. AT the same time a negative control (40 µL) containing 1× HF Buffer and 1 ng μL^{-1} YIpBB5 was treated equally. 2 µL and 5 µL of *Dpn*I-treated MEGAWHOP reaction was used to transform commercial heat shock competent *E. coli* NEB5α (High Efficiency, New England Biolabs, USA) according to the suppliers instructions. Transformants were selected on solid LB-medium supplemented with 100 mg L^{-1} ampicillin. Both transformations generated ca. 7700 cfu mL^{-1}. 5 µL of the negative control did not result in any transformants. Five additional transformation reactions of NEB5α were performed generating a total *E. coli* library of 57,370 ± 3261 cfu. These transformants were inoculated in 100 mL of LB-medium supplemented with 100 mg mL^{-1} of ampicillin and grown for 16 h at 37°C. The resulting culture was aliquoted in 25% glycerol and stored at -80°C. Part of the culture was used to purify and sequence the plasmids. This confirmed that all mutations were present in the plasmid mix (Fig. S2).

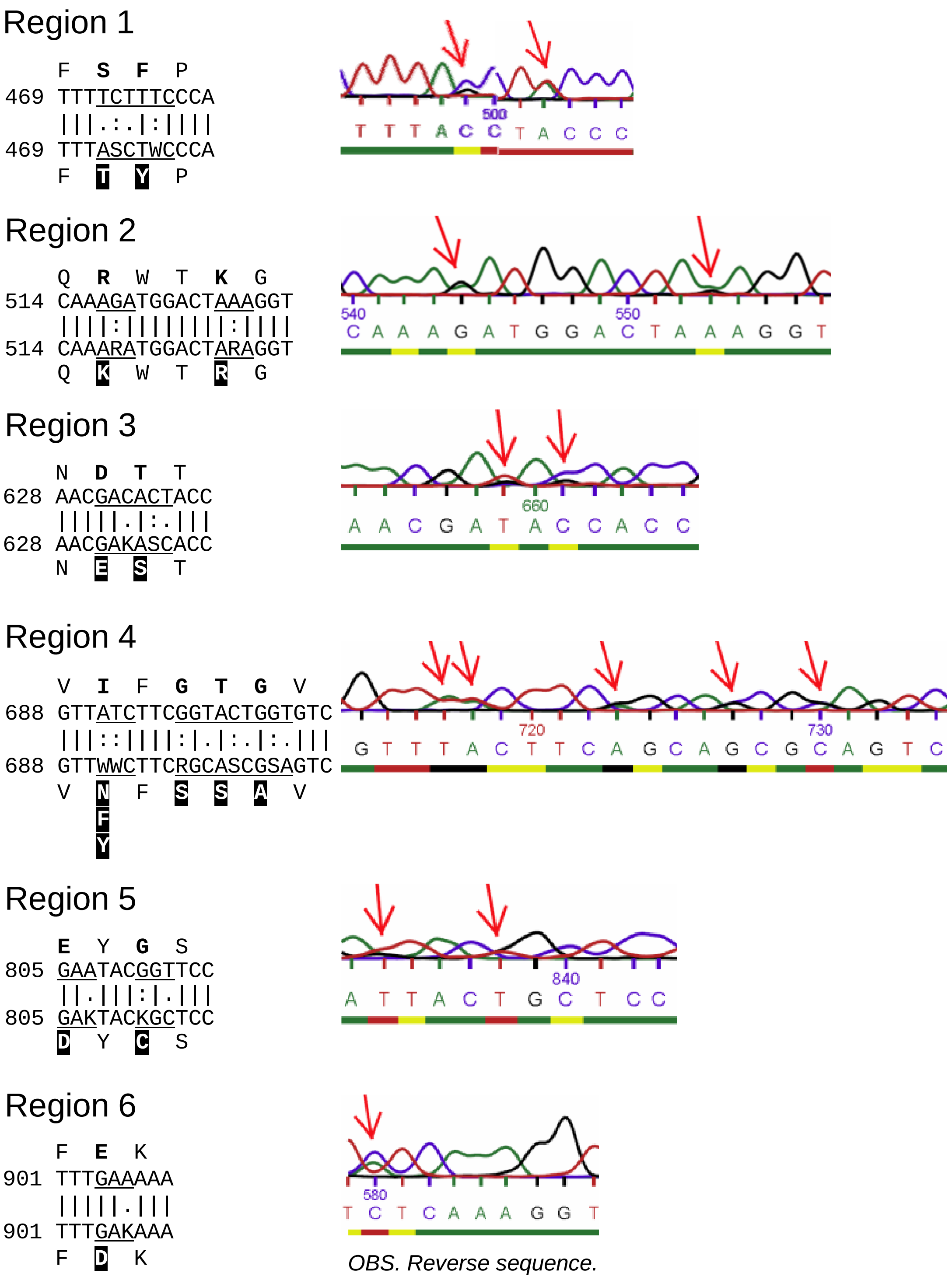

Supporting Figure S2. Sequencing of the *HXK2*-library

The nucleotide sequences of the mutated regions are shown to the left. The top sequence is the native sequence and the bottom sequence contains the introduced degeneracy. Codons that were modified are underlined and the native and alternative amino acid residues are indicated with bold and white-on-black letters, respectively. The right panel shows the corresponding region from the electropherogram (note that Region 6 is shown as the reverse complement). Arrows indicate the point of the degeneracy and the dual signals show that the mutations are indeed introduced in the megaprimer.

Construction of strains TMB3460, TMB3461 and TMB3462

Unless stated otherwise the PCR reactions contained 1× HF Buffer, 0.2 mM of each dNTP, 0.01 U μL^{-1} Phusion Hotstart DNA Polymerase II and 0.5 μM each of forward and reverse primers (Table S4). The reaction volume was 50 μL. OE-PCR reactions contained 200 fmol of each fragment. DNA fragments amplified by PCR were purified from 0.8% agarose gel using the QIAquick Gel Extraction Kit (Qiagen, Germany) unless stated otherwise.

Verification of correct integration and gene deletion was performed using the primers listed in Table S5 and the following reaction mix: 1× DreamTaq Buffer, 0.2 mM of each dNTP, 0.3 μM each of forward and reverse primers and 1 U μL^{-1} DreamTaq DNA Polymerase.

Construction of TMB3460 (*hxk2*-Δ)

The upstream and downstream fragments flanking the *HXK2* gene were amplified from genomic DNA from *S. cerevisiae* CEN.PK2-1C using the following PCR program: 30 s denaturation at 98°C, 30 cycles of 10 s denaturation at 98°C, 30 s annealing at 65°C (-0.5°C $cycle^{-1}$), 15 s extension at 72°C and 1 cycle of 10 min extension at 72°C.

The auxotrophic marker cassette *TRP1* was amplified from plasmid p424 using the following PCR program: 30 s denaturation at 98°C, 30 cycles of 10 s denaturation at 98°C, 30 s annealing at 65°C (-0.5°C $cycle^{-1}$), 1 min extension at 72°C and 1 cycle of 10 min extension at 72°C.

The deletion cassette HXK2_US-*TRP1*-HXK2_DS was created by OE-PCR using the following PCR program: 30 s denaturation at 98°C, 16 cycles of 10 s denaturation at 98°C, 30 s annealing at 68°C (-0.5°C $cycle^{-1}$) and 1 min extension at 72°C. At this point the reaction was halted at 4°C until primers HXK2_US_f and HXK2_DS_r had been added after which the program continued: 30 s denaturation at 98°C, 20 cycles of 10 s denaturation at 98°C, 30 s annealing at 63°C (-0.4°C $cycle^{-1}$) and 1 min extension at 72°C. The program was finished with a final 10 min extension at 72°C.

The amplified deletion cassette was purified and used to transform TMB3042. Transformants were selected on solid YNB medium with 2% glucose, 50 mg L^{-1} uracil and 220 mg L^{-1} leucine. Correct integration was verified by PCR amplification from chromosomal DNA of randomly selected colonies using primers HXK2_823US_f and TRP1_71_r. One positive clone was named TMB3460.

Construction of TMB3461 (*hxk2*-Δ *hxk1*-Δ*1*)

The upstream and downstream fragments flanking the *HXK1* gene were amplified from genomic DNA from *S. cerevisiae* CEN.PK2-1C. The auxotrophic marker cassette *URA3* was amplified from plasmid p426. All fragments were amplified using the following PCR program: 30 s denaturation at 98°C, 30 cycles of 10 s denaturation at 98°C, 30 s annealing at 65°C (-0.5°C $cycle^{-1}$), 1 min s extension at 72°C and 1 cycle of 10 min extension at 72°C.

The deletion cassette HXK1_US-*URA3*-HXK1_DS was created by OE-PCR using the following PCR program: 30 s denaturation at 98°C, 16 cycles of 10 s denaturation at 98°C, 30 s annealing at 68°C (-0.5°C $cycle^{-1}$) and 1 min extension at 72°C. At this point the reaction was halted at 4°C until primers HXK1_US_f and HXK1_DS_r had been added after which the program continued: 30 s denaturation at 98°C, 20 cycles of 10 s denaturation at 98°C, 30 s

annealing at 67°C (-0.4°C $cycle^{-1}$) and 1 min extension at 72°C. The program was finished with a final 10 min extension at 72°C.

The amplified deletion cassette was purified and used to transform TMB3460. Transformants were selected on solid YNB medium with 2% galactose and 220 mg L^{-1} leucine. Correct integration was verified by PCR amplification from chromosomal DNA of randomly selected colonies using primers HXK1_1510US_f and URA3_120_r. One positive clone was named TMB3461.

<u>Construction of TMB3462 (*hxk2*-Δ *hxk1*-Δ*1* *glk1*-Δ)</u>
The upstream and downstream fragments flanking the *GLK1* gene were amplified from genomic DNA from *S. cerevisiae* CEN.PK2-1C. The antibiotic marker cassette LoxP-*KanMX*-LoxP was amplified from plasmid pUG6. All fragments were amplified using the following PCR program: 30 s denaturation at 98°C, 30 cycles of 10 s denaturation at 98°C, 30 s annealing at 67°C (-0.5°C $cycle^{-1}$), 1 min extension at 72°C and 1 cycle of 10 min extension at 72°C.

The deletion cassette GLK1_US-[LoxP-*KanMX*-LoxP]-GLK1_DS was created by OE-PCR using the following PCR program: 30 s denaturation at 98°C, 16 cycles of 10 s denaturation at 98°C, 30 s annealing at 68°C (-0.5°C $cycle^{-1}$) and 1 min extension at 72°C. At this point the reaction was halted at 4°C until primers GLK1_US_f and GLK11_DS_r had been added after which the program continued: 30 s denaturation at 98°C, 20 cycles of 10 s denaturation at 98°C, 30 s annealing at 68.5°C (-0.4°C $cycle^{-1}$) and 1 min extension at 72°C. The program was finished with a final 10 min extension at 72°C.

The amplified deletion cassette was purified and used to transform TMB3461. Transformants were selected on solid YNB medium with 2% galactose, 220 mg L^{-1} leucine and 200 mg L^{-1} geneticin. Correct integration was verified by PCR amplification from chromosomal DNA of randomly selected colonies using primers GLK1_1050US_f and kanMX_32_r. One positive clone was named TMB3462.

Investigation of the glucose phosphorylating activity in strain TMB3462 revealed a significant level of activity despite confirmation of all deletion cassettes. Amplification of each gene using specific primers (Table S5) revealed that *HXK1* was still present in the genome (Fig. S3).

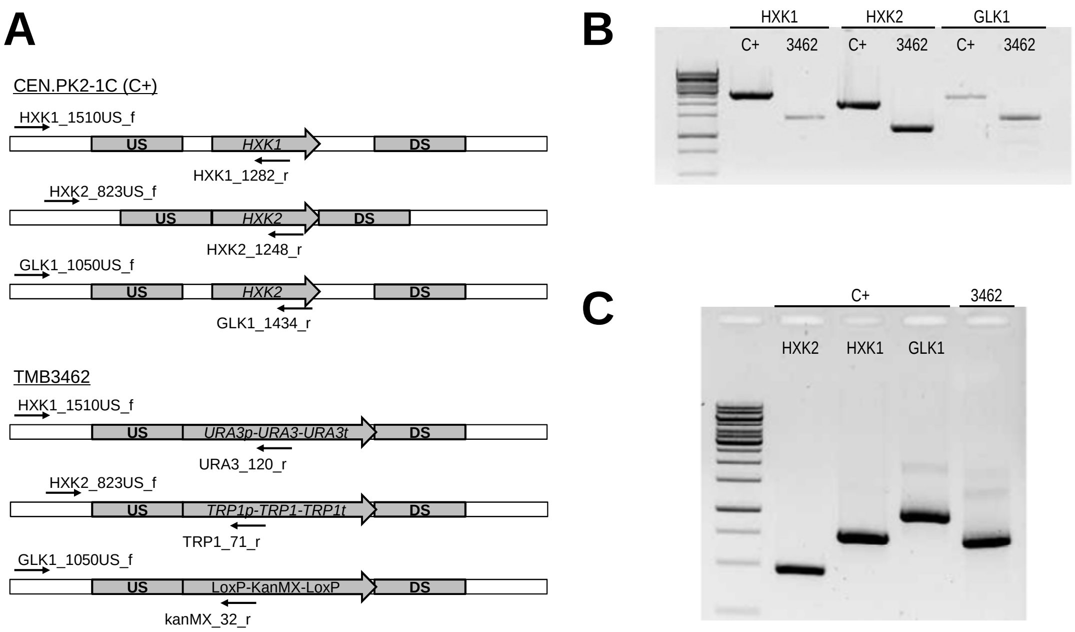

Supporting Figure S3. Verification results of integration and gene deletion.
A) Primers used with template from CEN.PK2-1C (positive control) and TMB3462. **B**) Amplification results of the set-up shown in **A**). **C**) Amplification of each gene using specific primers listed in Table S5. The reaction with template from TMB3462 contained primers for all genes.

Construction of the screening strain TMB3463

Generation of plasmid pUG62AUR

To facilitate the use of pUG6AUR, a new multiple cloning site was created near the LoxP site upstream of the aureobasidin A resistance gene using linkers. These linkers consisted of two long oligonucleotides, 37 and 35 bases, respectively, and were complimentary except for the sticky ends. These were instead complementary to the restriction sites *Sal*I and *Nde*I present in the pUG6AUR plasmid. All in all, the linkers included six different restriction sites: *Sal*I, *Kpn*I, *Sma*I, *Sph*I, *Avr*II and *Nde*I (Fig. S4).

```
Linker oligomers:
5'-TCGACCTGAGGTACCCCGGGCATGCATCCTAGGTGCA   -3'
3'-    GGACTCCATGGGGCCCGTACGTAGGATCCACGTAT-5'
   SalI      KpnI  SmaI SphI    AvrII   NdeI
```

Restriction sites:

SalI	5'..G^T C G A C..3'<br>3'..C A G C T^G..5'	SmaI	5'..C C C^G G G..3'<br>3'..G G G^C C C..5'	AvrII	5'..C^C T A G G..3'<br>3'..G G A T C^C..5'
KpnI	5'..G G T A C^C..3'<br>3'..C^C A T G G..5'	SphI	5'..G C A T G^C..3'<br>3'..C^G T A C G..5'	NdeI	5'..C A^T A T G..3'<br>3'..G T A T^A C..5'

Supporting Figure S4. Illustration of the 39 bases long linker used to create a multiple cloning site into the pUG6AUR plasmid.

The linker oligomers were annealed to each other by mixing 500 pmol of each oligonucleotide with 6 µmol NaCl in 1× DreamTaq Buffer (final volume 30 µL) and incubated at 100°C in a Thermocycler for 5 min. The temperature was then lowered slowly at 1°C min^{-1} until a temperature of 4°C was reached. The salt was removed through alcohol precipitation. 1 mL 99.5 % ethanol was added to the 30 µL mix and incubated at -80°C for 10 min. The tube was centrifuged for 12 min at 4°C and the supernatant was discarded. The pellet was washed once with 500 µL of 70% ethanol after which the pellet was dried in room temperature for 10 min. The small pellet was finally dissolved in 25 µL TE buffer giving an estimated concentration of 20 µM (ca. 475 ng μL^{-1}).

The annealed linkers were ligated to pUG6AUR, previously digested with *Sal*I and *Nde*I, in a 20 µL reaction containing the following: 1× Fast Digest Green Buffer, 50 ng μL^{-1} linker, 12.5 ng μL^{-1} linear pUG6AUR, 5% (w v^{-1}) PEG 4000, 0.5 mM ATP and 0.125 U μL^{-1} T4 DNA Ligase. The ligation mixture was incubated at room temperature for 60 min after which the ligase was inactivated at 70°C for 5 min. 2 µL of the ligation mix was removed and diluted to a final concentration of 2.5 ng plasmid μL^{-1}. 5 µL of the diluted ligation mix was used to transform heat shock competent *E. coli* NEB5α. Transformants were selected on LB-medium with 100 mg L^{-1} ampicillin. Successful ligation was determined by digesting the purified plasmids with *Kpn*I/*Sph*I or *Kpn*I/*Avr*II which are specific for the presence of the linker. The resulting plasmid was named pUG62AUR (Fig. S5).

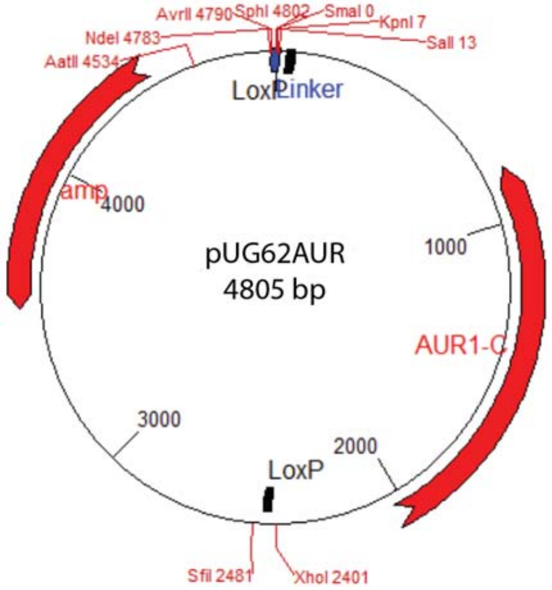

Supporting Figure S5. The pUG62AUR plasmid with a multiple cloning site consisting of the *Avr*II, *Sph*I, *Sma*I and *Kpn*I restriction sites.

<u>Construction of TMB3463 (*hxk2-Δ hxk1-Δ1 glk1-Δ hxk1-Δ2*)</u>

Unless stated otherwise the PCR reactions contained 1× GC Buffer, 0.2 mM of each dNTP, 0.01 U μL^{-1} Phusion Hotstart DNA Polymerase II and 0.5 µM each of forward and reverse primers (Table S6). The reaction volume was 50 µL. DNA fragments amplified by PCR were purified from 0.8% agarose gel using the QIAquick Gel Extraction Kit (Qiagen, Germany) unless stated otherwise.

Verification of correct integration and gene deletion was performed using the primers listed in Table S7 and the following reaction mix: 1× DreamTaq Buffer, 0.2 mM of each dNTP, 0.3 μM each of forward and reverse primers and 1 U μL^{-1} DreamTaq DNA Polymerase.

The additional upstream and downstream fragments flanking the *HXK1* gene were amplified from genomic DNA from *S. cerevisiae* CEN.PK2-1C using the following PCR program: 30 s denaturation at 98°C, 5 cycles of 10 s denaturation at 98°C, 15 s annealing at 60.9°C, 8 s extension at 72°C, 25 cycles of 10 s denaturation at 98°C, 20 s annealing and extension at 72°C and 1 cycle of 10 min extension at 72°C.

The purified upstream and downstream fragments were digested with *Sph*I/*Avr*II and *Kpn*I/*Sph*I, respectively. The digested fragments were ligated into pUG62AUR, previously digested with *Kpn*I/*Avr*II, using a 3:1 molar ratio of insert to vector and an incubation time of 2 h at room temperature. 5 μL of the ligation mix was used for transformation of heat shock competent *E. coli* NEB5α. Transformants were selected on LB-medium with 100 mg L^{-1} ampicillin. Successful ligation was determined by colony PCR using primers HXK1_DS2_f and HXK1_US2_r and the resulting plasmid was named pUG62AUR-HXK1USDS (Fig. S6).

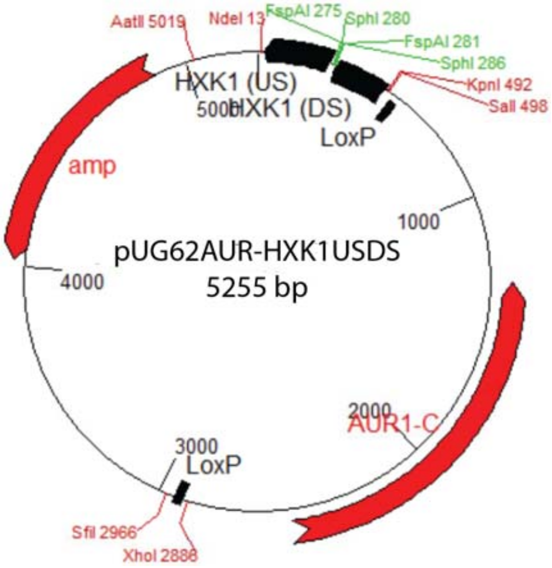

Supporting Figure S6. The pUG62AUR-HXK1USDS plasmid containing homologous regions flanking the *HXK1* gene.

TMB3462 was transformed with pUG62AUR-HXK1USDS, linearized with *Fsp*I, according to High efficiency protocol. Before plating the transformants the cells were resuspended in 1 mL of YP with 2% galactose and incubated at 30°C for 2.25 h. Transformants were selected on solid YNB medium containing 2% galactose, 220 mg L^{-1} leucine and 0.15 mg L^{-1} aureobasidin A. Correct integration was verified by colony PCR on randomly selected colonies using primers HXK1_US_f and AmpR-R130. Attempts to amplify the *HXK1* gene using primers HXK1_547_f and HXK1_1282_r did not result in any amplification, indicating the gene had been deleted. This was confirmed by a very low glucose phosphorylating activity in one positive clone which was named TMB3463 (Fig. S7).

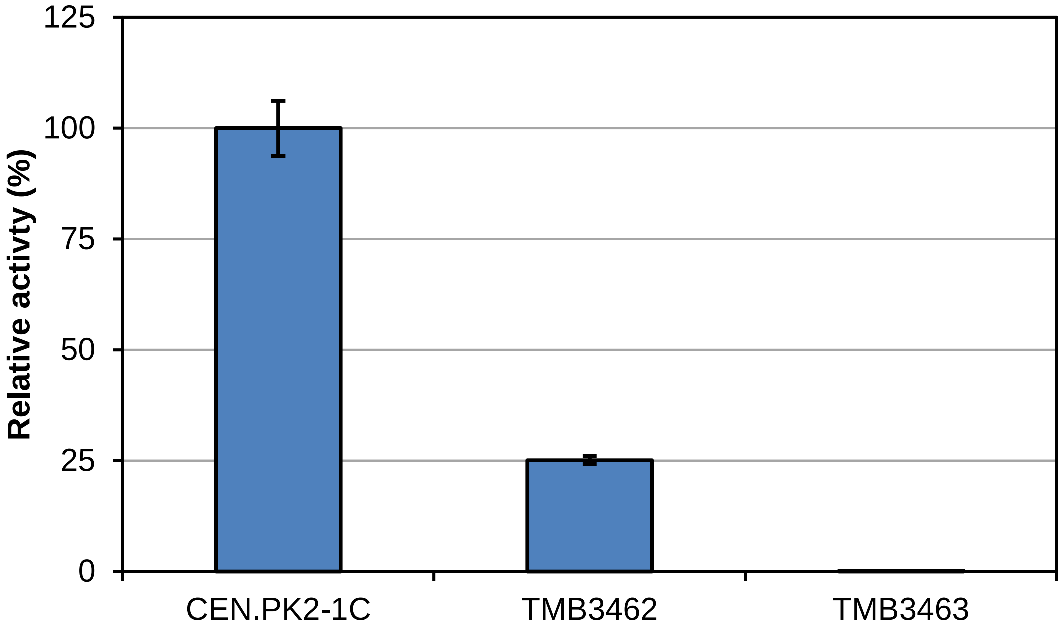

Supporting Figure S7. The glucose phosphorylating activity in strains TMB3462 (3Δ) and TMB3463 (4Δ) relative to the wild-type CEN.PK2-1C strain.

Supporting Tables

Supporting Table S1. Maximum specific consumption rates, production rates and yields in anaerobic batch fermentation of 20 g L^{-1} glucose and 50 g L^{-1} xylose by TMB3492 (Hxk2p-wt) and TMB3493 (Hxk2p-Y).

Values are given as mean ± standard deviation of two independent experiments.

	**Glucose phase**		**Xylose phase**	
	**TMB3492**	**TMB3493**	**TMB3492**	**TMB3493**
μ_{max} (h^{-1})	0.35±0.02	0.34±0.01	0.013±0.000	0.027±0.004
**Specific rates (mmol g CDW^{-1} h^{-1})**				
$r_{max,glc}$	-20.6±3.2	-21.8±0.4	–	–
$r_{max,xyl}$	–	–	-3.48±0.35	-5.72±0.99
$r_{max,xylt}$	–	–	0.909±0.017	0.960±0.014
$r_{max,glyc}$	6.31±0.49	6.61±0.42	0.085±0.041	0.052±0.037
$r_{max,ac}$	1.04±0.02	1.22±0.09	0.040±0.021	0.028±0.005
$r_{max,etoh}$	39.1±5.6	41.3±2.5	1.60±0.40	1.34±0.98
**Yields (g g sugar^{-1})**				
$Y_{xylt/s}$	–	–	0.261±0.007	0.270±0.003
$Y_{glyc/s}$	0.129±0.005	0.127±0.007	0.028±0.001	0.027±0.000
$Y_{ac/s}$	0.015±0.002	0.015±0.000	0.009±0.003	0.008±0.002
$Y_{etoh/s}$	0.398±0.012	0.392±0.036	0.251±0.003	0.255±0.009
$Y_{X/s}$	0.078±0.005	0.070±0.002	0.056±0.013	0.055±0.008

Supporting Table S2. Overall yields and production rates in anaerobic batch fermentation of 20 g L^{-1} glucose and 50 g L^{-1} xylose by TMB3492 (Hxk2p-wt) and TMB3493 (Hxk2p-Y).

Values are given as mean ± standard deviation of two independent experiments.

**Overall yields (g g total sugar^{-1})**			**Overall production rates (g L^{-1} h^{-1})**		
	**TMB3492**	**TMB3493**		**TMB3492**	**TMB3493**
$Y_{xylt/s}$	0.144±0.010	0.158±0.002	q_{xylt}	0.133±0.007	0.139±0.000
$Y_{glyc/s}$	0.061±0.008	0.059±0.002	q_{glyc}	0.057±0.008	0.052±0.003
$Y_{ac/s}$	0.011±0.002	0.012±0.001	q_{ac}	0.010±0.002	0.010±0.001
$Y_{etoh/s}$	0.258±0.004	0.262±0.004	q_{etoh}	0.240±0.000	0.231±0.001
$Y_{X/s}$	0.032±0.005	0.036±0.003	q_X	0.029±0.004	0.031±0.002

Supporting Table S3. Primers used to construct the mutated *HXK2* megaprimer.

Name	Sequence (with wobble positions in bold letters)
**Degenerate primers**	
Group_1_f	5'-CATTGGGTTTCACCTTTA**S**CT**W**CCCAGCTTCTCAAAAC-3'
Group_1_r	5'-GTTTTGAGAAGCTGGG**W**AG**S**TAAAGGTGAAACCCAATG-3'
Group_2_f	5'-GAAGGTATCTTGCAAA**R**ATGGACTA**R**AGGTTTTGATATTCC-3'
Group_2_r	5'-GGAATATCAAAACCT**Y**TAGTCCAT**Y**TTTGCAAGATACCTTC-3'
Group_3_f	5'-GTTGCTTTGATAAACGA**K**A**S**CACCGGTACTTTGGTTG-3'
Group_3_r	5'-CAACCAAAGTACCGGTG**S**T**M**TCGTTTATCAAAGCAAC-3'
Group_4_f	5'-GAAACTAAGATGGGTGTT**WW**CTTC**R**GCA**S**CG**S**AGTCAATGGTGCTTAC-3'
Group_4_r	5'-GTAAGCACCATTGACT**S**CG**S**TGC**Y**GAAG**WW**AACACCCATCTTAGTTTC-3'
Group_5_f	5'-CAATGGCCATCAACTGTGA**K**TAC**K**GCTCCTTCGATAATGAAC-3'
Group_5_r	5'-GTTCATTATCGAAGGAGC**M**GTA**M**TCACAGTTGATGGCCATTG-3'
Group_6_f	5'-CAGGCCAACAAACCTTTGA**K**AAAATGTCTTCTGGTTAC-3'
Group_6_r	5'-GTAACCAGAAGACATTTT**M**TCAAAGGTTTGTTGGCCTG-3'
**Additional primers**	
Yip128-F1	5'-GGCCTTTTGCTGGCCTTTTG-3'
Yip128-R1	5'-AAGGGGGATGTGCTGCAAGG-3'

Supporting Table S4. Primers used to construct deletion cassettes.

Name	Sequence
HXK2_US_f	5'-GGTACCTAGAAATGGCTATCATGC-3'
HXK2_US_r	5'-TATATATAGTAATGTCGTTTATTTAATTAGCGTACTTATTATGTGTGG-3'
HXK2_DS_f	5'-GATAGGGTTGAGTGTTGTTACTTAATTTGTAAATTAAGTTTGAACAACAAG-3'
HXK2_DS_r	5'-AGAAGAATCCACGCGTAAAAATCG-3'
TRP1_f	5'-TAAGTACGCTAATTAAATAAACGACATTACTATATATATAATATAGGAAG-3'
TRP1_r	5'-CTTAATTTACAAATTAAGTAACAACACTCAACCCTATCTAGGTC-3'
HXK1_US_f	5'-TGGCGTGGGGTGGGGTGATT-3'
HXK1_US_r	5'-GTTAAGCCAGCCCCGACACCGGGCACGTGCGGGAGTTT-3'
HXK1_DS_f	5'-GTCTATCAGGGCGATGGCCCGCGACCCAGATTGCAGAAGATCCC-3'
HXK1_DS_r	5'-AGGTGCCCTTGCTAGCAT-3'
URA3_f	5'-AAACTCCCGCACGTGTGCCCGGTGTCGGGGCTGGCTTAAC-3'
URA3_r	5'-GGGATCTTCTGCAATCTGGGTCGCGGGCCATCGCCCTGATAGAC-3'
GLK1_US_f	5'-AGAGGAGGCGAGCAGCAGGG-3'
GLK1_US_r	5'-GTCGACCTGCAGCGTACGAAAGTGCCCACCGTTTGAGCGT-3'
GLK1_DS_f	5'-ATCAGATCCACTAGTGGCCTATGCGACAGCCTCGCCCTCTTCCGT-3'
GLK1_DS_r	5'-CGGGCAGTGCAGTGTGAGGG-3'
loxPKanMX_f	5'-ACGCTCAAACGGTGGGCACTTTCGTACGCTGCAGGTCGAC-3'
loxPKanMX_R	5'-ACGGAAGAGGGCGAGGCTGTCGCATAGGCCACTACCTCTTCCGT-3'

Supporting Table S5. Primers used to confirm correct integration and gene deletion.

Name	Sequence	Application
HXK2_823US_f	5'-ACCACACGCATGCCTTCATTCC-3'	Chromosomal integration
TRP1_71_r	5'-GCGGCCTCTGTGCTCTGCAA-3'	
HXK2_773_f	5'-TTCCACCATCTGCTCCAATGGC-3'	Gene deletion
HXK2_1248_r	5'-TGCAGCAATGTGACCGGTCTTG-3'	
HXK1_1510US_f	5'-AGCGGTTCGCTTCCAGCACC-3'	Chromosomal integration
URA3_120_r	5'-GGTGGTACGAACATCCAATGAAGCA-3'	
HXK1_547_f	5'-GTCGAAGGCCACGATGTCGTCC-3'	Gene deletion
HXK1_1282_r	5'-CCCTTAGCGGCGGCTTCCTT-3'	
GLK1_1050US_f	5'-ACGGCGACAGCCGGTTGGCTT-3'	Chromosomal integration
kanMX_32_r	5'-CGCGGCCTCGAAACGTGAGT-3'	
GLK1_433_f	5'-CCGGACGAGTTGGCCAAGGG-3'	Gene deletion
GLK1_1434_r	5'-CCTCTCACCCTCGGCACCCA-3'	

Supporting Table S6. Primers used to amplify additional fragments upstream and downstream the *HXK1* gene.

Restriction sites are indicated in bold.

Name	Sequence	Restriction site
HXK1_US2_f	5'-TAG**GCATGC**GCATTGGTACCTTAGGACCGTTGAG-3'	*Sph*I
HXK1_US2_r	5'-CG**CCTAGG**GATTGAGTTGTTTGGGTGAGTTTG-3'	*Avr*II
HXK1_DS2_f	5'-ACT**GGTACC**TTGGTCTTCTTCATGCATCATTTCA-3'	*Kpn*I
HXK1_DS2_r	5'-TTG**GCATGC**ATCAGCTATAAGAGACGAAATTGCT-3'	*Sph*I

Supporting Table S7. Primers used to confirm correct integration of pUG62AUR-HXK1USDS and *HXK1*-gene deletion.

Name	Sequence	Application
HXK1_1510US_f	5'-AGCGGTTCGCTTCCAGCACC-3'	Chromosomal integration
AmpR-R130	5'-AATGATACCGCGAGACCCAC-3'	
HXK1_547_f	5'-GTCGAAGGCCACGATGTCGTCC-3'	Gene deletion
HXK1_1282_r	5'-CCCTTAGCGGCGGCTTCCTT-3'	
